# Supplementary material for: Differential richness inference for 16S rRNA marker gene surveys
Source: Genome Biol. 2022 Aug 1;23:166. doi: 10.1186/s13059-022-02722-x (PMC9344657; doi:10.1186/s13059-022-02722-x)
Supplement: Supplementary file 1 — Additional file 1. Presents the theoretic models for substitution errors introduced during amplification and sequencing, incorporating back mutations. The influence of recovered abundance-dependent accumulation of false sequencing read classifications is characterized. Related figures and literature references are contained within the note. [file 13059_2022_2722_MOESM1_ESM.pdf]

Additional File 1 : The type I error rate of sequence similarity thresholds.

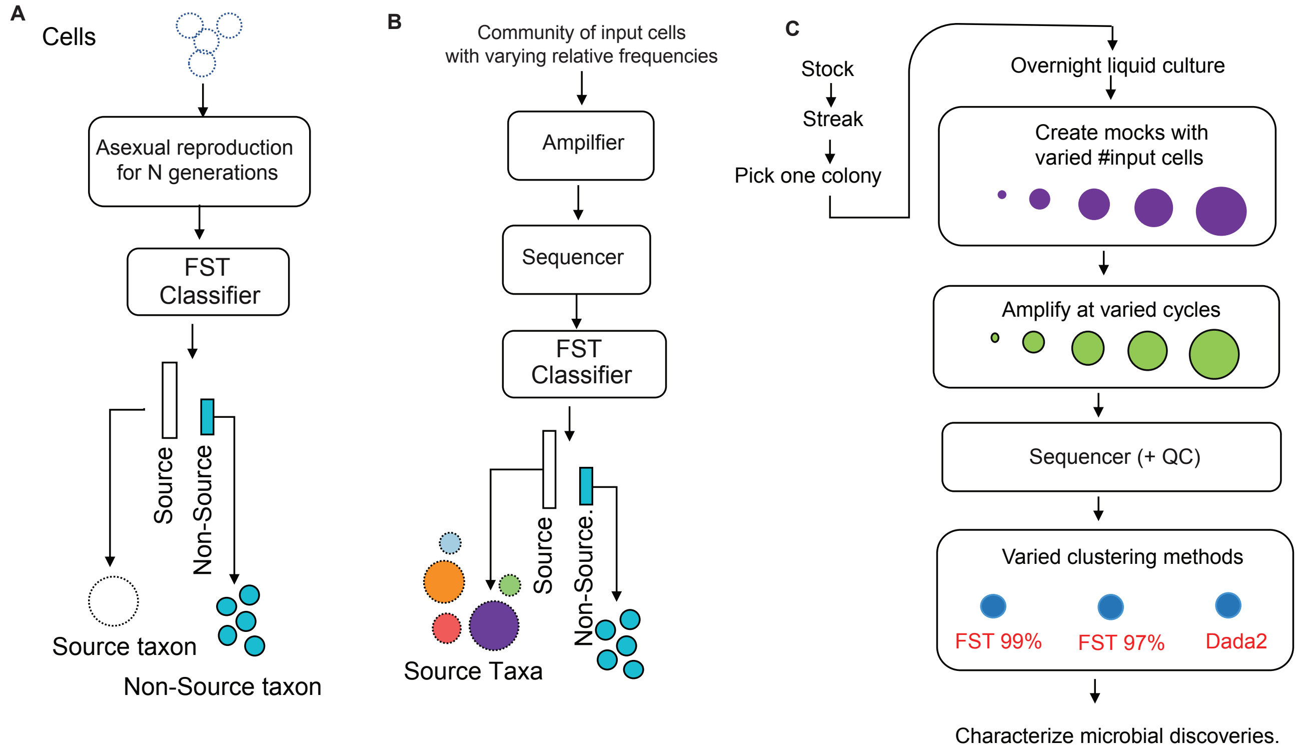

Fig. S1: Questions asked in sections 0.1 - 0.2 and main text section 2.1. When an apriori fixed sequence similarity threshold (FST) based classifier is input a source (reference) sequence, and a set of its noisy realizations from (A) cellular reproduction-like processes, the type I misclassification errors transform to false microbial discoveries through sequence clustering. We quantify the type I classification error rate. In (B), we seek the same for a community of sequences input to a chain of amplification and sequencing processes. In (C), we aim to experimentally study false discovery accumulations in a 16S survey of single colony derived bacterial culture (main text). The laboratory samples are constructed by varying two experimental parameters (number of input cells and 16S rRNA amplification cycles), and a bioinformatics parameter, the microbial population reconstruction approach.

Given a query and a reference sequence of the same length L, we quantify the sequence similarity between the two as the number of sites where they differ in nucleotide identitities. We use the term $FST(\delta)$ to denote a binary sequence classifier that classifies a query as a source type (i.e., reference) when the sequence similarity between the query and source is $\leq \delta L$. Thus, $\delta = .01$ and $\delta = .03$ correspond to 99% and 97% sequence similarity thresholds respectively.

Fig. S1 outlines a subset of questions asked in this paper. Briefly, given a reference DNA segment and a well-defined set of its noisy realizations as input, we seek the type I error rate of a fixed sequence similarity threshold based classifier $FST(\delta)$. The set of erroneous variants are generated thorugh nucleotide substitutions from (A) asexual cellular reproduction, (B) polymerase chain reaction induced amplification, (C) sequencing machine and (D) a chain of amplification and sequencing processes. In each case, the cardinality of the generated variant set dictates the total number of classifications, and hence the number of type I errors generated. The type I errors generated through these mechanisms go onto organize as false microbial discoveries in a study via the clustering algorithm used.

0.1 Type I error rate of an FST classifier when genotyping natural variation induced by cell growth

Consider a selectively neutral DNA segment of length L in a cell undergoing perfect cell divisions for N generations, leading to 2^N daughter cells (Fig. S2). Let p_m be the mutation probability per base per cell division regardless of the type, and assume that the mutations occur independently across sites. Every daughter cell can inherit a subset of substitutions from its parent cell, can mutate a subset of substitutions back to their ancestral states and can generate new substitutions through mutations. The resulting phylogeny is represented as an inhomogeneous Markov process on a binary tree of height N. The root represents the mother cell, the leaves represent the L length DNA segments in the 2^N daughter cells, and a cell is to its two daughter cells with edges. Let $S_1^1 \ldots S_{2^N}^N$ denote the random number of substitutions in the child segments. The internal nodes in the tree represent the various ancestral forms sampled along the way.

Under these modeling assumptions, the following results ensue. Specific mathematical details are given in **Appendix** section **1.1**. The average substitutions per child for each level n is obtained as $\langle s^{(n)} \rangle = 3p_m L \left[1 + \sum_{a=2}^{n} \prod_{b=a}^{n} \left(\tilde{p}_{inh}^{(b)} - 3p_m\right)\right]$, which is absolutely convergent in n. Here $\tilde{p}_{inh}^{(n)} \to .75(1 - p_m)$ as $n \to \infty$. For any level n of the tree, the probability distribution over substitutions, $p(S_i^{(n)})$, is identical for all children $i = 1 \ldots 2^n$. Post N generations, when a child is input to the $FST(\delta)$ classifier, it operates with a type I error rate of

$$q_{asexual}^{(N)}(\delta) = p(S_1^{(N)} > \delta L) \tag{1}$$

When processing all children, this error rate leads to $2^N \cdot q_{asexual}^{(N)}(\delta)$ number of type I errors, on average. When the initial population consists of B_0 cells *identical* in the the L length DNA region, each undergoing independent exponential growth spurts for N generations, a forest of B_0 such binary trees operate, leading to an average number of type I errors of:

$$B_0 \cdot 2^N \cdot q_{asexual}^{(N)}(\delta) \tag{2}$$

Generalization to base-type and position specific mutation rates can be made (**Appendix** section **1.1.1**) but the qualitative results remain the same: beyond amplification cycles, false classification errors grow with the initial population size.

Calculations for the Pseudomonas dilution experiment A single cell dividing once every ~ 30 minutes, when grown for ~ 12 *hours*, would pass ~ 24 generations. For a sequenced 16S hypervariable segment (e.g., V12) length of $250bp$, mutation rate of $\sim 10^{-8}$ per base per cell division, analytic calculations using our cell division model above leads to an average 7.5×10^{-6} substitutions in the DNA segment per child (**Appendix** eqn. (11)). For a 99% sequence similarity threshold, probability of generating an OTU-type child genotype (i.e., deviates by $> \delta L$ substitutions), computed numerically, was: 7.49×10^{-6}, leading to a total of ~ 0.0077 misclassified children. For a 97% sequence similarity threshold, these numbers are essentially equal to zero (less than machine precision).

0.2 Type I error rate of an FST classifier for sequencing molecules post- amplification and sequencing

Amplification Amplification via polymerase chain reaction deviates from exponential cell growth in at least one significant fashion. Here, the template DNA strands from the previous generations coexist with newly created strands in every generation, and hence in the final amplification product. So molecules and even their individual strands can vary substantially in the number of extension/mutation cycles they were a product of (**Fig. S3**). This induces varying substitution probabilities across molecules. Results from the previous section are straightforwardly utilized to derive the following results.

Consider a single DNA molecule of length L, amplified for N cycles. As before, we restrict to substitution errors alone. We assume mutations are independent across sites, denoting by p_m the per nucleotide mutation probability per base per generation regardless of the type. Each newly generated strand derives its own set of substitutions by inheriting a subset from its template strand and/or newly generating them de novo.

First, assume that the PCR reaction happens with 100% efficiency leading to perfect doubling reactions every cycle. The number of substitutions expected in a template strand that is a result of n extension cycles is the same as that of the previous asexual reproduction case. For all strands that were a product of n extension cycles, the probability distribution over substitutions $\alpha(S_i^{(n)})$ is identical. The type I error rate when classifying a template strand that is a product of n extension cycle is:

$$q_\alpha^{(n)}(\delta) = \alpha(S_1^{(n)} > \delta L)$$

When the FST classifier is input all the strands from the amplification product, the average number of type I errors resulting is $2\sum_{n=1}^{N}\binom{N}{n}q_\alpha^{(n)}$. Here, we have exploited the Moore & Moranas's result [1] that the number of template strands that are a product of n extension cycles is $2\binom{N}{n}$. When there are B_0 initial molecules, each undergoing independent perfect doubling reactions for N generations, we obtain:

$$B_0 \cdot 2\sum_{n=1}^{N}\binom{N}{n}q_\alpha^{(n)} \tag{3}$$

type I errors on average. The average number of substitutions observed in the amplification product at generation m is: $\sum_{n=1}^{m}\frac{\binom{m}{n}}{2^n}\langle s^{(n)}\rangle$, with $\langle s^{(n)}\rangle$ defined in the previous section.

When the amplification is imperfect, with imperfection quantified by λ ($\lambda = 1$ implying 100% efficiency), the average number of type I errors is given, upto first order, as:

$$B_0 \cdot 2\sum_{n=1}^{N}\lambda^n\binom{N}{n}q_\alpha^{(n)} \tag{4}$$

where we have used Pritchard et al.,'s result that the average number of strands which underwent n extension cycles under imperfect amplification is $2\binom{N}{n}\lambda^n$ [2].

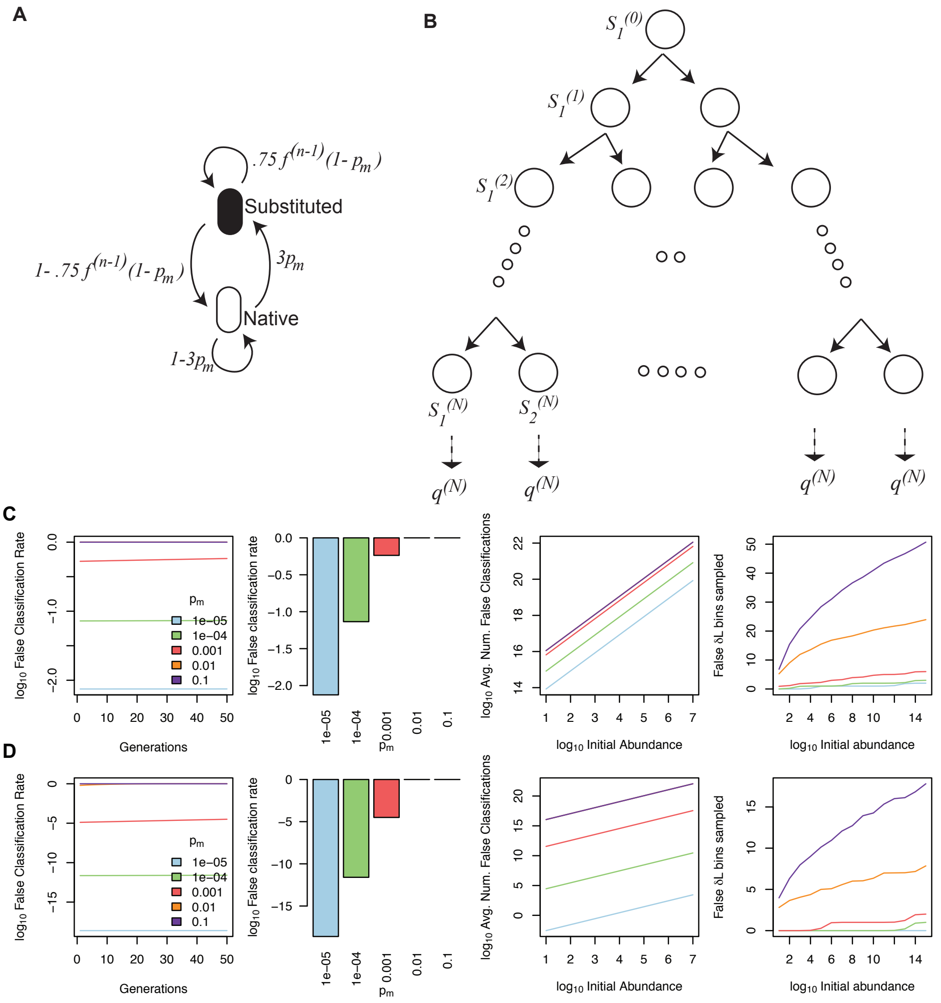

Fig. S2: Cellular reproduction $\rightarrow$ clustering. Collect the same L length DNA segment from all daughter cells post cell growth for a fixed number of replications N, and input to the binary FST classifier as queries, along with the corresponding ancestral mother segment as the reference. We seek the type I error rate of falsely classifying a daughter cell's segment as non-source i.e, not of the mother type. Such non-source type segments go on to generate false OTU varieties. (A) Transition probabilities among substituted and native states of a nucleotide during growth cycle n, when the per nucleotide mutation rate is p_m. $f^{(n)} \rightarrow 1$ as $n \rightarrow \infty$. (B) Binary tree representing perfect doubling of a cell's DNA segment for N cycles. $S_i^{(n)}$ denotes the realized number of substitutions at daughter cell i in level n of the tree. Each leaf-node on the tree represents one of the final 2^N daughter cells' DNA segments resulting from cell growth. $q(N)$ is the probability that a daughter cell (generated naturally) is an OTU-type. $S_i^{(n)}$ evolves according to the process outlined. (C) The false classification rates of an $FST(\delta = .01)$ classifier for various per base per generation mutation rates across generations (left) and at 50 generations (second from left). Average number of artifactual OTU-type daughters (i.e., those that deviate greater than 1% from the mother sequence) due to substitution errors grow with the number of mother cells ("initial abundance") (third from left). Bin sequences that are $k \times (\delta L)$ distance away for $k = 1 \ldots 1/(\delta L)$. Bins contribute to new false microbial discoveries through new sequence clusters. Each bin can form multiple clusters as sequences that are $k \times \delta \times L$ distances apart from the source sequence, can themselves be δL distance apart. An increase in number of such bins correspond to an increased number of false microbial discoveries. We plot the expected number of such bins sampled from 99% and 97% similarity threshold based clustering of reads for various per base per generation mutation rates (fourth from left). (D) Same as (C) but for 97% sequence similarity threshold.

Sequencing The position-specific quality scores output by the sequencing machine for each sequencing read offers a standard strategy to model sequencing induced substitution probabilities directly. Briefly, quality scores can be transformed to error probabilities conditional on the observed nucleotide, and further integrated to obtain net position-wise substitution probabilities. An inhomogeneous Poisson process parameterized by these probabilities is used to model the total number of substitutions occurring in a sequencing read. The type I error rate is obtained as the probability that the machine induced substitutions exceed the FST threshold as $q_\psi(\delta)$.

$$\begin{aligned}\psi(S=s) &= \int \left[\sum_{x \in \binom{L}{s}} \prod_{k:x(k)=1} p(k) \prod_{k:x(k)=0} (1-p(k)) \right] dH \\ q_\psi(\delta) &:= \psi(s > \delta L)\end{aligned} \tag{5}$$

where H is the distribution function of substitution probability profiles underling sequencing read generation, as obtained from the quality score profiles. The inner sum runs over all possible L length s substituted sequences, k runs over their substituted positions, and $p(k)$ represents the probability of substitution at the site obtained with a suitable transformation from a sequencing machine's quality score profiles.

The expected number of type I errors, when sequencing at a depth of τ is then:

$$\tau \cdot q_\psi(\delta) \tag{6}$$

Chaining amplification and sequencing The aforementioned more exact model for sequencing induced substitutions is cumbersome to model a chain of amplification and sequencing effects. We make a Poisson approximation to the sequencing error model, which models a constant substitution probability across all sites by the estimated average. We found the approximation to have a low error rate ($\approx 10^{-5}$, based on Serfling [3]) both because of the overall weak per base substitution probabilities by design and because of quality score aware trimming of reads performed by analysts.The latter stabilizes error variance across positions. These simplifications allow a tractable computation of $\psi(s|r)$ (**Appendix** section 1.6), the probability that the sequencing machine outputs an s substituted sequence when input an already r substituted variant from amplification. For the moment, assume amplification efficiencies are comparable across all input sequences and also that there are no further technical biases entering the reaction. Under these conditions, the substitution probabilities of the amplification-sequencing chain are obtained per input molecule as:

$$\begin{aligned}p(S=s) &= \sum_{r=0}^{L} \alpha(r)\psi(s|r) \\ q_{\alpha\to\psi}(\delta) &:= p(S > \delta L).\end{aligned} \tag{7}$$

When sequenced at a depth τ, this leads to an expected $\tau \cdot q_{\alpha\to\psi}(\delta)$ number of false classifications.

Generalizing to a community of input sequences So far we have restricted ourselves to a contrived system, in which the input to our amplification-sequencing pipeline consisted solely of a single source sequence at some initial abundance. We slightly generalize and consider a community of true input source sequences $i = 1 \ldots p$, each with an (apparent) input relative frequency η_i. It is very reasonable to assume that sequencing machine generates reads in amounts proportional to their input concentrations (see [4, 5]). The relative abundances of source sequences enter the analysis through the following model: $Y_i|\tau \sim Multinomial(\tau, \eta_i)$, where τ is the sequencing depth and η_i is the (apparent) input relative

abundance (ref. eqn 2, [5]). Then the net error rate in eqn. (7) can be viewed as a result of integration of input sequence-wise conditionals.

$$q_{\alpha\to\psi}(\delta) = \sum_i \eta_i \cdot q_{\alpha\to\psi}(\delta) := \sum_i q^{(i)}_{\alpha\to\psi}(\delta) \tag{8}$$

Thus, the expected type I errors resulting from each source sequence are predicted as a line along the expected recovered abundance of a source sequence i :

$$q^{(i)}_{\alpha\to\psi}(\delta)\cdot\tau = q_{\alpha\to\psi}(\delta)\cdot\eta_i\cdot\tau = q_{\alpha\to\psi}(\delta)\cdot EY_i \tag{9}$$

Based on eqn. 1 in Kumar et al., 2018 [5], it is straightforward to see varied amplification efficiences across input sequences and the presence of further technical biases alter these equations but the overall qualitative result remains the same: (i) Beyond sample depth, the *apparent* input frequencies (i.e., input frequencies altered by technical biases) is a key determinant of false discoveries. (ii) The false classifications grow as a function of recovered abundance, which upto technical biases, is multiplicative in input abundance and sample depth.

The type I errors made above go on to generate new taxa discoveries algorithmically based on their sequence configurations and abundances. Tracking these microbial discoveries as they continue to aggregate algorithmically from a single source sequence, let alone a mixture of sequences, is difficult. We leave this as an open question, settle for obtaining a rough idea on the scale of such discoveries in **Appendix section 1.2** and proceed to experimentally characterize them in the single colony experiment described in the main text.

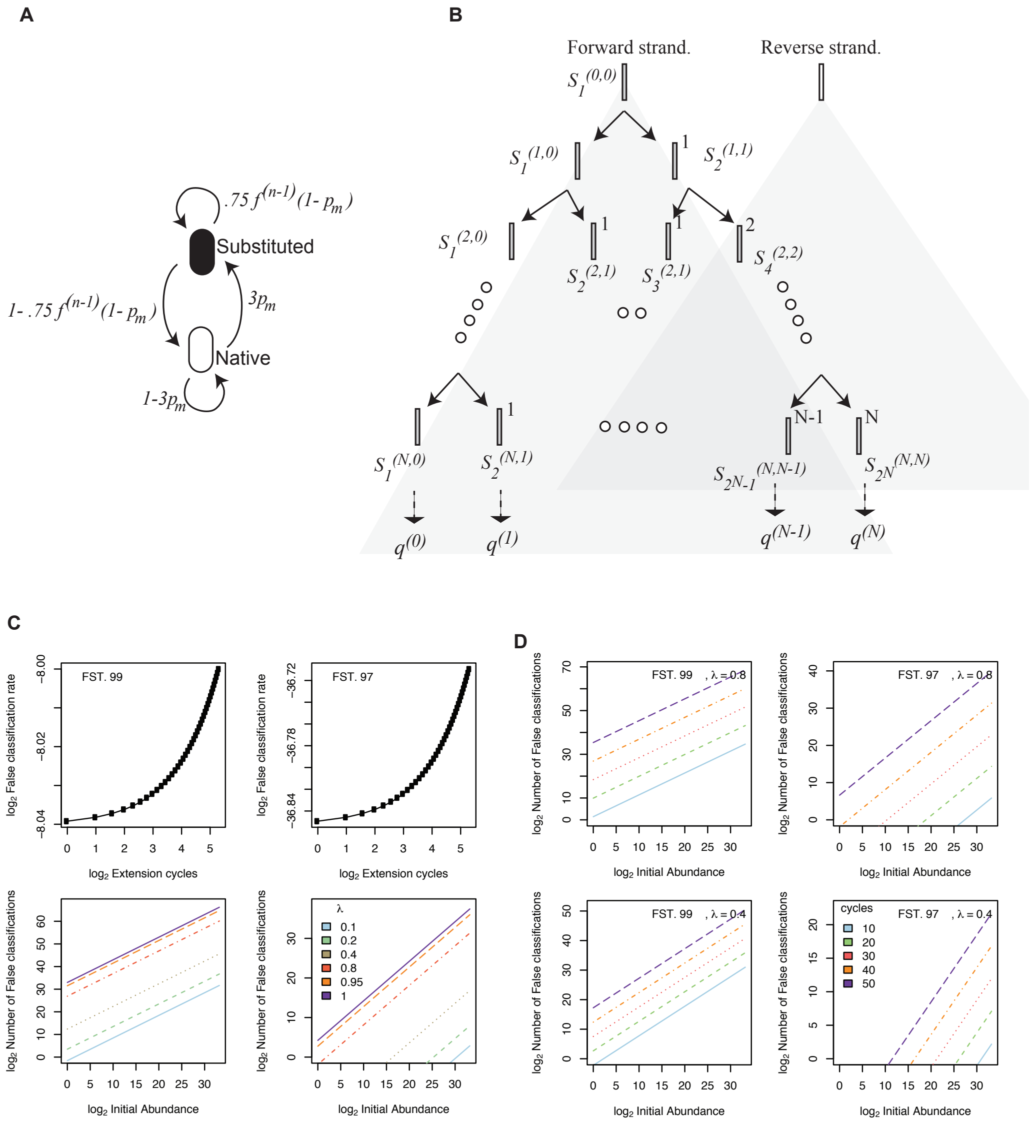

Fig. S3: Amplification → clustering (A) Transition probabilities among substituted and native states of a nucleotide during amplification cycle n, when the per nucleotide mutation rate is p_m. $f^{(n)} \to 1$ as $n \to \infty$. (B) A binary tree representing perfect doubling of a forward strand for N cycles. $S_i^{(n,m)}$ denotes the number of substitutions of a product strand at level n of the tree, while being a product of m amplification cycles. Each leaf-node on the tree represents one of the final 2^N child DNA molecules' forward strand product resulting from amplification. $q(N)$ is the probability that a product strand resulting from N cycles of amplification is an OTU-type. $S_i^{(n,m)}$ evolves according to the process outlined. (C) (top row) For two FST threshold settings (99% left column, 97% right column), calculated false classification rate for various extension cycles. Taq polymerase's per nucleotide mutation rate of 1.2×10^{-4} per base per doubling, post 40 PCR cycles. (bottom row) Correspondingly, the average number of artifactual OTU-type strands in the amplification product, generated due to substitution errors, grow with the initial concentration ("input abundance"). (D) Rows correspond to two different amplification efficiencies (80% and 40%). In each case, we plot the total number of false classifications. Columns correspond to two different decision rules for the binary classifier: 99% vs 97% sequence similarity. For $\lambda < 1$, we use a first order approximation to calculate the expected behavior of the classifier.

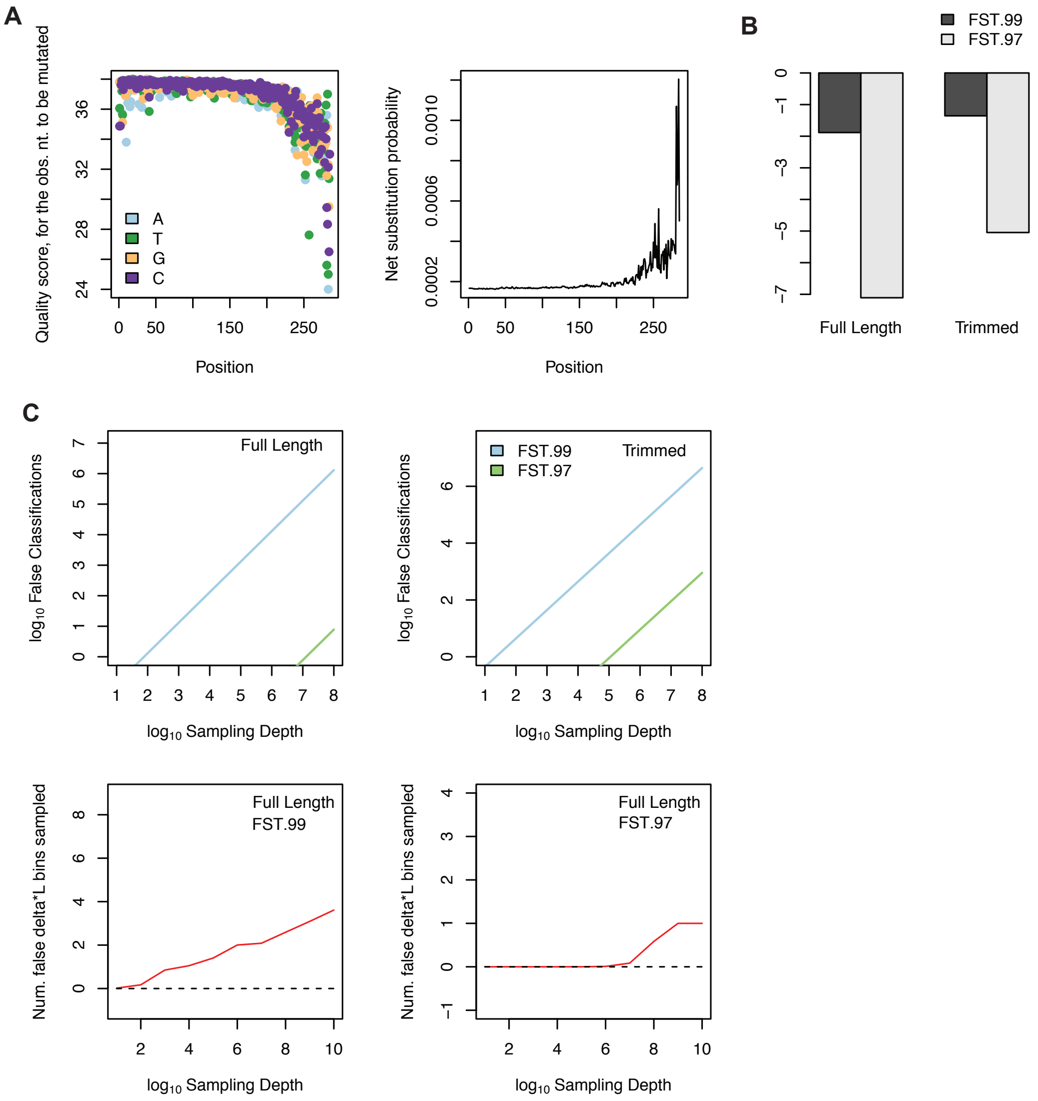

Fig. S4: Sequencing → clustering. (A) (left) Average quality score profiles derived for the various nucleotide observations and (right) average error probability derived from quality score profiles. (B)Expected false classification rates for 99% and 97% sequence similarity threshold based classifiers, when read trimming is restricted to first 10 bases alone ("full length"), and an additional 85 bases from the end ("trimmed"). The false classification rate is lower for the heavier trim setting. This is because the distance threshold of δL is more relaxed (higher). (C) (top row) the expected number of false classifications are visualized as a function of sampled abundance for full length and trimmed settings, at 99% and 97% sequence similarity thresholds. (C) (bottom row) The expected number of false δL bins sampled when clustering with 99% and 97% sequence similarity, as a function of sampling depth, for the full length setting.

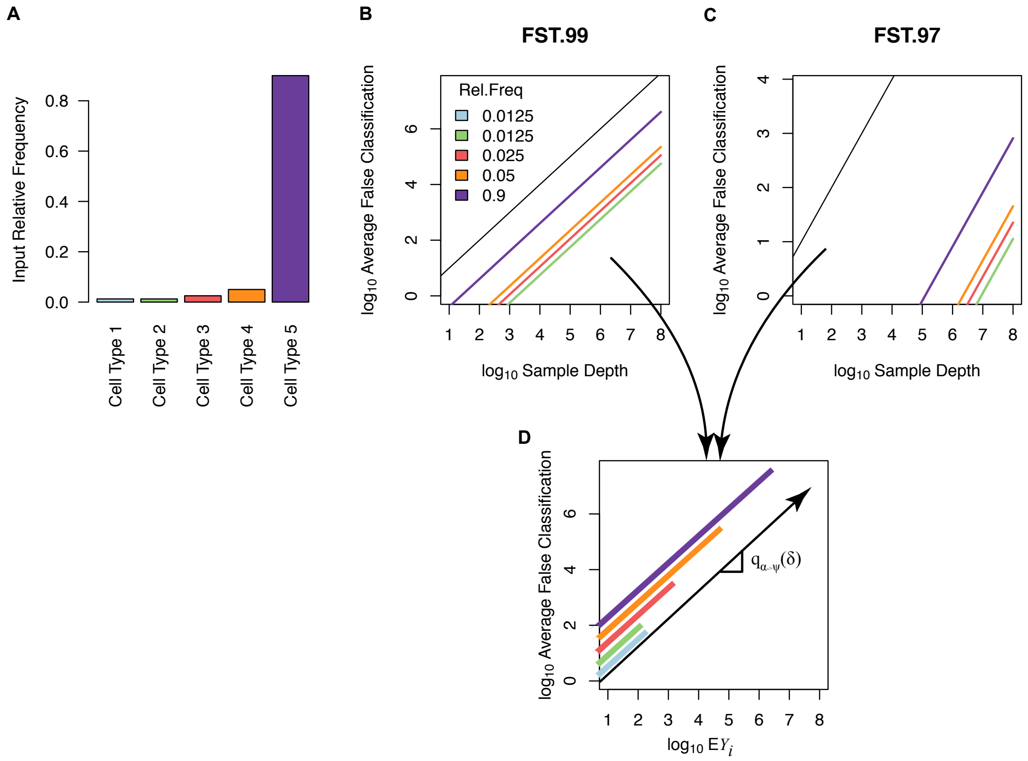

Fig. S5: Amplification→ Sequencing → clustering chain. Given each source sequence and the set of sequencing reads emitted from a chain of amplification and sequencing processes, the FST classifier decides if the read is source or not based on a fixed sequence similarity threshold. (A)For a community of species with varied input relative frequencies, expected number of false sequencing read classifications are presented as a function of sampling depth for (B) 99% and (C) 97% sequence similarity thresholds respectively. (D) As indicated by the supplementary eqn. (9), the expected number of false classifications for the entire community are captured by two parameters: the expected output abundance of each true source type ($EY_i, i = 1 \ldots 5$ for the five species indicated), and a slope parameter that is the false classification rate, which subsumes the various biochemical and bioinformatics parameters of an amplification-sequencing-clustering chain. The colored bars represent the region along the black line sampled by the colored true input sequences. Amplification and sequencing parameter settings same as in supplementary Figs. S3 and S4.

1 Appendix

1.1 Type I error rate of FST classifiers when genotyping natural variation

Model Consider a branching process where a single cell divides asexually for N generations, leading to 2^N descendants (**Fig.2B**). Each of the 2^n nodes at any level n of the binary tree represents a distinct DNA molecule generated by replicating the ancestral molecule length L for n generations. In addition to assuming perfect doubling, we assume the following: First, we assume mutations occur independently along the entire length of the DNA sequence. Second, we let p_m be the mutation probability for every nucleotide, regardless of its type $\{A,T,G,C\}$. Finally, we shall assume all locations in the sequence suffer the same mutation rate.

At any level n, the nodes are indexed left to right on the tree with a subscript $i \in 1,\dots 2^n$ and a superscript (n). Let the random variable $S_i^{(n)} \in \{0,1,\dots L\}$ specify the total number of substitutions generated at node i in level n of the tree. Let $z_i^{(n)}$ indicate the parent node (in level $n-1$) of child node i at level n in the tree. Conditioned on the binary tree, the parent-child relationships $z_i^{(n)}$ is deterministic. For simplicity in notation below, we will be implicit about conditioning on the binary tree (and therefore, $z_{\cdot}^{(\cdot)}$), p_m, L and the source DNA sequence whose amplification is considered.

Consider the process:

$$
\begin{aligned}
S_1^{(0)} &= 0, \text{ number of substitutions at root node is 0} \\
S_i^{(n)}|X_{i1}^{(n)},X_{i2}^{(n)} &= X_{i1}^{(n)} + X_{i2}^{(n)} \; \forall \, i = 1\dots 2^n \text{ nodes}, \; n = 1\dots N \text{ levels} \\
X_{i1}^{(n)}|(S_{z_i^{(n)}}^{(n-1)} = s) &\sim Binomial(s, \tilde{p}_{inh}^{(n)}), \\
X_{i2}^{(n)}|(S_{z_i^{(n)}}^{(n-1)} = s) &\sim Binomial(L-s, 3p_m)
\end{aligned}
\tag{10}
$$

In words, the substitution number at the root node, because it corresponds to that of the source sequence, is set to 0. For every non-root node at level n, its substitution number $S_i^{(n)}$ is realized by conditioning on the substitution number of its parent node $S_{z_i^{(n)}}^{(n-1)}$ as follows. Given the assumption that the mutations happen independently, the two Binomial random variates $X_{i1}^{(n)}$ and $X_{i2}^{(n)}$ respectively capture the inherited substitutions at a per nucleotide rate of $\tilde{p}_{inh}^{(n)}$ and newly injected substitutions (from non-substituted nucleotides in parent) at a per nucleotide rate of $3p_m$ (**Fig.1A**, and **Appendix 1.3**). The probability of inheritance of a substitution is derived as: $\tilde{p}_{inh}^{(n)} = .75 \cdot (1-p_m) \cdot \left[1-(1-4p_m)^{n-1}\right] = .75 \cdot (1-p_m) \cdot f^{(n-1)}$ where $f^{(n-1)} \to 1$ as $n \to \infty$. For large n, the substitution inheritance probability settles at $.75 \cdot (1-p_m)$. The probability of inheritance is a function of the generation number n because per site parental marginals for being a substitution varies with the number of generations passed since the ancestral state.

Expected substitution numbers It is interesting to ask for the expected number of substitutions accumulated in each of the 2^n product DNA strings after amplification cycle n. It is equaly interesting to ask the same question when the amplification itself is driven for a large number of cycles.

The following results appear in **Appendix 1.4**.

(1) The expected number of substitutions is found to be identical for all nodes at a given level n, and *scales with* L as:

$$
\begin{aligned}
E[S_i^{(n)}] &= 3p_m \cdot L \cdot \left[1 + \sum_{a=2}^{n} \prod_{b=a}^{n} \left(\tilde{p}_{inh}^{(b)} - 3p_m\right)\right] \\
&= E[S_j^{(n)}] \; \forall j = 1\dots 2^n]
\end{aligned}
\tag{11}
$$

(2) $\lim_{n\to\infty} E[S_i^{(n)}]$ is well defined and is trapped by:

$$\lim_{n\to\infty} E[S_i^{(n)}] \in \left(3p_m L, \; \frac{3p_m L}{1-\left(\tilde{p}_{inh}^{(n)} - 3p_m\right)}\right) \tag{12}$$

Fig. S6 illustrates the nature of these results for two p_m values. Here the exact averages are calculated numerically and are plotted alongside their upper bound. We see that the average subtitution numbers are strongly dependent on p_m. It pays a laboratory to invest in high quality DNA polymerase regardless of the number of amplification cycles run. It is also observed that the average substitution numbers per cycle quickly approach their asymptotic averages. The upper boundary is a rather tight approximation for the expectations.

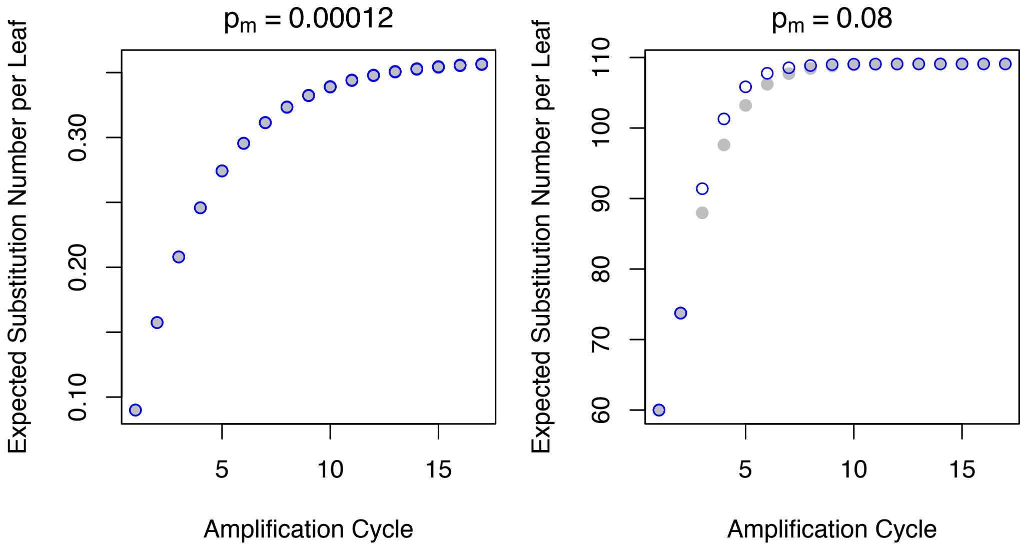

Fig. S6: Evolution of average substitution numbers is plotted for two per nucleotide mutation rate values (p_m). $L = 250$. $p_m = 1.2 \times 10^{-4}$ is the laboratory measured mutation rate for the *Taq* polymerase. Exact substitution numbers per node (Eqn. (11)) is plotted as filled gray circles as a function of amplification cycle n. The respective upper bounds are plotted as open blue circles.

Probability distribution over substitutions It is shown that for a given level n, the probability distribution over substitutions is *identical* for all nodes at that level (**Appendix 1.5**). This distribution is given as:

$$p(S_i^{(n)} = s_c) = \sum_{s_p=0}^{L} p\left(S_i^{(n)} = s_c | S_{z_i^{(n)}}^{(n-1)} = s_p\right) \cdot p\left(S_{z_i^{(n)}}^{(n-1)} = s_p\right)$$

$$\text{and, } p(S_i^{(n)}) = p(S_j^{(n)}) \text{ for all } j = 1\ldots 2^n, \text{ and for all } n = 1\ldots N \tag{13}$$

The distribution is available numerically given the parameters p_m, L and N.

Type I error rate of an FST classifying the children of an ancestral cell We now answer one of the central questions of this article: Among all the 2^N leaf-nodes generated naturally via asexual reproduction, how many are expected to be classifed as non-source type by an FST classifier? It is these classifications that will go on to generate false microbial discoveries as new OTUs. The probability that the ith leaf node is an OTU is the probability that it accumulates a

substitution number $> \delta L$. This is given as :

$$q_{asexual}^{(N)} := p(S_i^{(N)} > \delta L) \tag{14}$$

Here the non-dependence of the notation $q_{asexual}^{(N)}$ on i arises because of eqn. (13). Define $T_i^{(N)}$ as the random variable indicating that the leaf node i in level N is a non-source type , and define $T_+^{(N)} = \sum_{i=1}^{2^N} T_i^{(N)}$. We seek $p(T_+^{(N)})$.

It is clear $E(T_i^{(N)} = 1) = q_{asexual}^{(N)}$. The expected total number of OTUs is given as:

$$E[T_+^N] = 2^N \cdot q_{asexual}^{(N)} \tag{15}$$

This result is for *per cell* undergoing exponential growth for N generations (i.e., per binary tree with 2^N leaf nodes) . If there are B_0 identical (identicalness, defined with respect to the L length DNA segment under consideration) copies of the same cell undergoing independent amplifications for the same number of N cycles, there are B_0 independent binary trees each generating $2^N q_{asexual}^{(N)}$ OTUs on average, leading to a total of $B_0 \cdot 2^N q_{asexual}^{(N)}$ expected. Thus we conclude that, under the model assumptions, the number of amplification-error induced "artifact" OTUs scale with initial bacterial abundance and amplification cycles. **Fig.1D** shows this behavior for a reasonable choice of parameters.

1.1.1 Generalizing for nucleotide specific mutation rates and position dependencies

The main goal is to relax two of the assumptions made in the simplified model above: a constant mutation rate (p_m) per-nucleotide regardless of the nucleotide type, and its location in DNA segment.

The results from the simplified model in the previous subsection is heavily utilized below. As before, the goal is to derive $p(S_i^{(N)})$ for a given length L sequence.

It is assumed that we know the two dimensional function $p_m(x,y)$, where x indicates the position $x = 1 \dots L$, and y indicates the nucleotide. Fix N. For every $x = 1 \dots L$ and for every $y \in \{A, T, G, C\}$, calculate $p(S_i^{(N)} = 1)(x,y)$ from the simplified model in eqn (13) setting $p_m = p_m(x,y)$ and a sequence length of 1 nucleotide. This yields the function $p(S_i^{(N)})(x,y)$. Consider any two L length sequences u and v, and let $v(x)$ retrieve the nucleotide type at position x of sequence v. The independence assumption among the sites allows the probability of generating v in a given leaf-node of the N cycle binary tree to be written as:

$$\begin{aligned} p(u \to v \; in \; a \; given \; leaf-node) &= \prod_{x:\; v(x) \; is \; substituted} p(S_i^{(N)})(x, v(x)) \prod_{h: v(h) \; is \; not \; substituted} p(S_i^{(N)})(h, v(h)) \\ \implies p(S_i^{(N)} = s) &= \sum_{i=0}^{\binom{L}{s}} p(u \to v_i) \end{aligned} \tag{16}$$

where v_i is an L length nucleotide string with s substitutions. This is the *Poisson-Binomial* distribution parameterized by the vector of probabilities $p(S_i^{(N)})(\cdot, v)$. The OTU generation probability per leaf-node $q_{asexual}^{(N)}$, and the expectation calculations retain the same form as in the simplified model before.

$$\begin{aligned} q_{asexual}^{(N)}(\delta) &:= p(S_i^N > \delta L) \\ E[T_+^{(N)}] &= 2^N \cdot q_{asexual}^{(N)}(\delta) \\ E[\text{OTU type daughters given initial abundance } B_0] &= B_0 \cdot 2^N \cdot q_{asexual}^{(N)}(\delta) \end{aligned} \tag{17}$$

1.2 From type I errors to false microbial discoveries

The type I errors made above go on to generate new taxa discoveries algorithmically based on their sequence configurations and abundances. Tracking these microbial discoveries as they continue to aggregate algorithmically from a single source sequence, let alone a mixture of sequences, is difficult. Consider the following example. There are $\binom{L}{x} \cdot 3^x$ possible nucleotide sequences that are x substitutions away from a given L length source sequence. However, these sequences are not all pairwise x substitutions apart, and hence, experimentally sampled subsets can get clustered in OTU clustering programs. We leave this as an open question and settle for obtaining a rough idea on the scale of such discoveries as a function of similarity thresholds δL.

Consider an isolated system with a single source sequence and its possible noisy realizations. The distance from the source sequence is binned in units of δL. The kth bin generates substitutions in the interval $((k-1) \times \delta L, k \times \delta L]$ with probability $p_k = F(k \cdot \delta L) - F((k-1) \cdot \delta L)$. Here F is the distribution function underlying substitutions. For $k = 1$, the left boundary is closed at 0. Sequence falling in the $k = 1$ th bin are subsumed as the source OTU. For the remaining δL width bins, both variation in the substitution positions and nucleotides in those positions create new taxa discoveries. We count only a subset of OTU possibilities as follows.

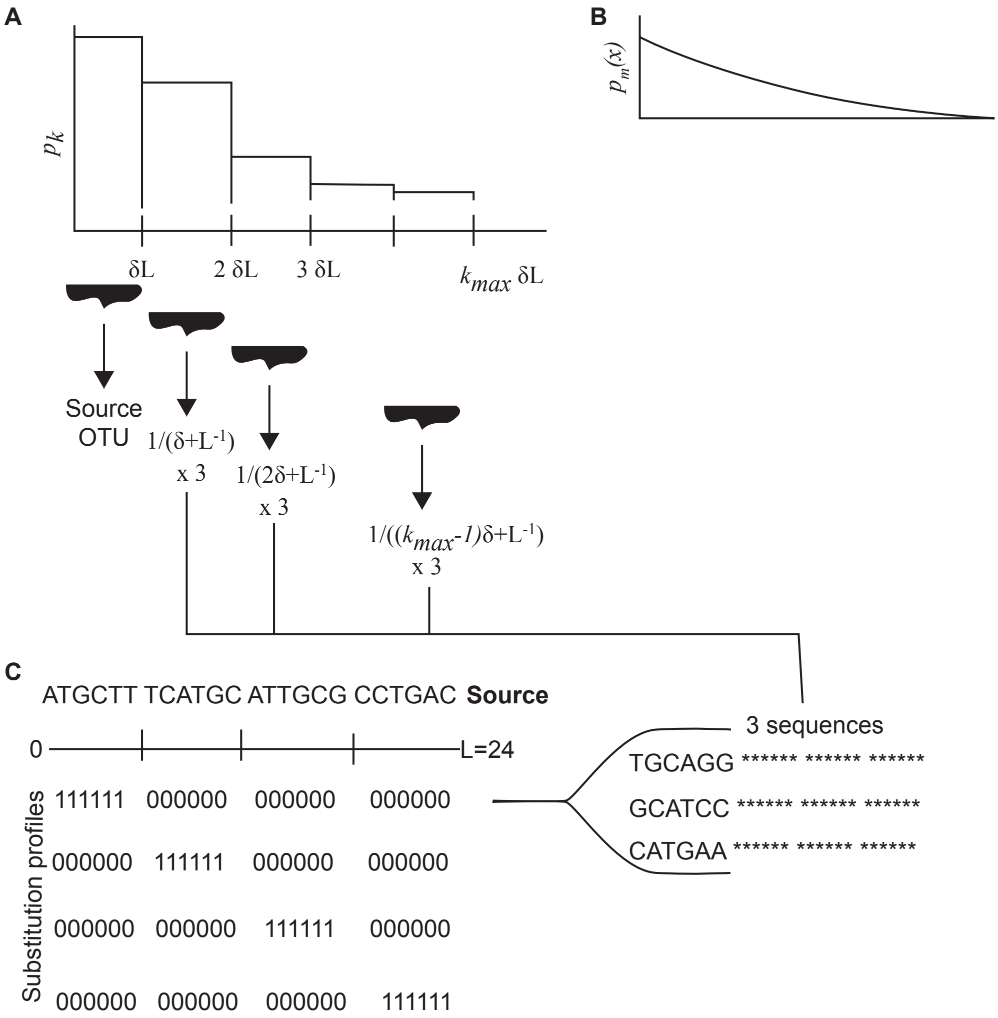

Fig. S7: From type I errors to false microbial discoveries. The L possible number of substiutions for a length L sequence is binned in δL widths. Substituted sequences falling in the first bin is clustered in to the source OTU. The net sampling probabilities for rest of the bins are determined by the cumulative distribution function underlying substitutions, which in turn is determined by the site specific mutation probabilities indicated in (B) as $p_m(x)$, where x is the position. (C) L length substitution profiles with δL substitutions in consecutive positions are shown. The total number of such profiles is indicated for each window by the arrows. Each such profile can give rise to three OTU centers δL distance away. The total such OTU centers, a subset of all possible OTU types, are obtained by summing along the bins.

First, as also predicted by the simplified models in the previous sections, it is very reasonable to expect the substitution sampling probabilities to fall with the number of subsitutitons, and hence, new OTU centers are likely to be picked from just beyond each bin's left boundary, on average. So consider the total number of substitutions $x \in \{\delta L+1, 2\delta L+1, \ldots, (k_{max}-1)\delta L+1\}$, where k_{max} is an integer chosen such that the probability of generating substitutions beyond $k_{max}\delta L$ is very small. An $L-$ length substitution profile is a bit string that indicates the positions of substitutions with a value 1. For each $x \in \{\delta L+1, 2\delta L+1, \ldots, (k_{max}-1)\delta L+1\}$, we construct a subset of substitution profiles as follows: divide the source sequence into $\frac{L}{x}$ non-overlapping windows. Correspondingly, generate L length $\frac{L}{x}$ substitution profiles with only the consecutive positions in the $x-$length window holding a value of 1. For any given $x-$ substitution profile, it is straightforward to see that there are 3 nucleotide sequences that are themselves pairwise x substitutions apart. When they occur, they are guaranteed to form distinct OTUs. These are the possible "center" sequences for each x. Thus, we have counted about $\frac{L}{x} \cdot 3$ OTUs for x substitutions. As an example, for $x = \delta L+1$, we have $3 \times \frac{1}{\delta + L^{-1}}$ OTU possibilities.

The total number of OTU possibilities within this restricted set of substitution profiles is then: $\sum_{k=1}^{k_{max}-1} \frac{1}{k\delta + L^{-1}}$. Following Yarza et al., [6] who recommend a $\sim 94.5\%$ sequence similarity clusters for genus reconstructions, we choose $k_{max}(\delta) = .055/\delta$. For $\delta = .01$ (i.e., 99% sequence similarity threshold), $L = 250$, this leads to about 512 artifactual OTUs. For $\delta = .03$ (i.e., 97% sequence similarity threshold), this leads to 90 OTUs. These numbers are one-half to one-third of the total number of taxa found in our experimental study in the next section.

We must note the caveats. We have only considered substitution errors. We have ignored sampling characteristics (not all the aforementioned OTUs would occur in an experiment), ignored further OTU possibilities from substitution profiles with nonconsecutive substitutions within a window, ignored further nucleotide variations within the kth δL window that can deviate δL away from the anchor strings chosen at $(k-1)\delta L + 1$, and ignored other ensuing complications when there are multiple true input source sequences to consider. Where this analysis can perhaps be reasonably justified is with a single source sequence and when sites have a non-uniform mutation rate. Sort the positions in the decreasing order left to right, such that the probability of substituting is highest on the left end and weakest on the right. On average then, sites accrue mutations left to right. When the probability of non-consecutive substitutions in this sorted space is sufficiently low, ignoring those less likely configurations has a lower impact on these calculations.

1.3 Derivation for substitution ($3p_m$) and substitution inheritance probabilities ($p_{inh}^{(n)}$)

Because there are 4 bases (A, T, G & C), a switch to a new nucleotide (i.e., a substitution) can occur with probability $3p_m$, and a nucleotide remains faithful to itself with probability $1 - 3p_m$. Hence, $X_{i2}^{(n)}$ is determined by a per nucleotide probability of $3p_m$. This result also enforces a natural constraint on p_m: $0 \leq 3_p m \leq 1 \implies p_m \in \left[0, \frac{1}{3}\right]$. In this work, we consider the interval $p_m \in \left(0, \frac{1}{3}\right)$ interesting.

On the other hand, the inheritance rate of a substitution as a substitution in the child node is determined both by the nucleotide specification of the substitution in the parent node and the mutation rates determining the inheritance of the same substituted nucleotide / a possible mutation of it to a different nucleotide state that still renders the location in a substituted state in the child node. This is formalized in the derivation below.

For concreteness, consider a single sequence of parent-child relationships represented in the binary tree. We can obtain one such sequence by fixing $i = 1$ in $S_i^{(0)} \to S_i^{(1)} \to S_i^{(2)} ... \to S_i^{(N)}$. So set $i = 1$ until otherwise noted. Let $o_i^{(n,l)}$ indicate the realized nucleotide alphabet at position l in node i on level n. Let $\xi_i^{(n,l)}$ indicate if the lth position in child node i at level n is a substitution. Consider the special case that the true nucleotide at the lth position in the original DNA sequence is $o_i^{(0,l)} = A$ (**Fig. S8**). For notation simplicity, we do not explicitly indicate conditioning for $o_i^{(0,l)} = A$ below. The question seeks:

$$\begin{aligned} p(\xi_i^{(n,l)} = 1 | \xi_i^{(n-1,l)} = 1, o_i^{(0,l)} = A) &= \sum_{b \in T,G,C} p(\xi_i^{(n,l)} = 1 | \xi_{z_i^{(n)}}^{(n-1,l)} = 1, o_{z_i^{(n)}}^{(n-1,l)} = b) p(o_{z_i^{(n)}}^{(n-1,l)} = b) \\ &= \sum_{b \in T,G,C} p(\xi_i^{(n,l)} = 1 | \xi_{z_i^{(n)}}^{(n-1,l)} = 1, o_{z_i^{(n)}}^{(n-1,l)} = b) p(o_{z_i^{(n)}}^{(n-1,l)} = b) \end{aligned} \tag{18}$$

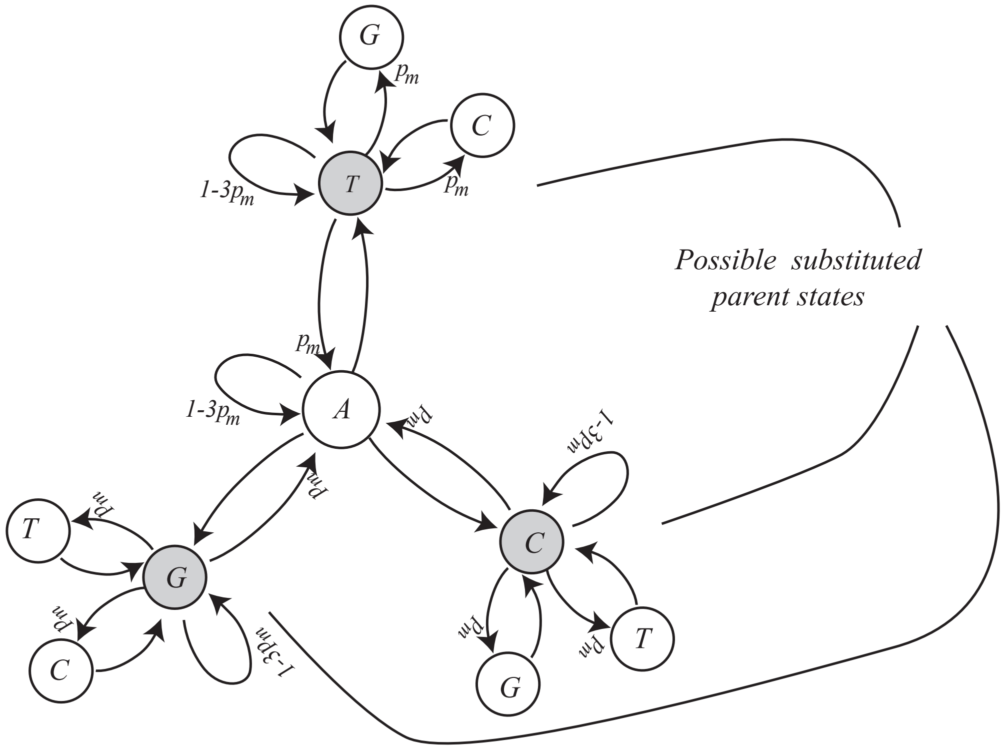

Fig. S8: Possible per-nucleotide substituted states for a parent-node when the true nucleotide state is an A. The transitions among nucleotides that retain a substituted state are shown and their rates identified. Transitions to the true nucleotide state and their probabilities are also described.

Expand each conditional in eqn. 18 as:

$$\begin{aligned} p(\xi_i^{(n,l)} = 1 | \xi_{z_i^{(n)}}^{(n-1,l)} = 1, o_{z_i^{(n)}}^{(n-1,l)} = T) &= p(T \to T) + p(T \to G) + p(T \to C) \\ &= (1-3p_m) + p_m + p_m \\ &= (1-3p_m) + 2p_m \\ &= (1-p_m) \end{aligned} \tag{19}$$

Similarly, obtain:

$$\begin{aligned} p(\xi_i^{(n,l)} = 1 | \xi_{z_i^{(n)}}^{(n-1,l)} = 1, o_{z_i^{(n)}}^{(n-1,l)} = G) = (1-p_m) \\ p(\xi_i^{(n,l)} = 1 | \xi_{z_i^{(n)}}^{(n-1,l)} = 1, o_{z_i^{(n)}}^{(n-1,l)} = C) = (1-p_m) \end{aligned} \tag{20}$$

For the second set of terms in eqn. (18), observe:

$$\begin{aligned} \text{For } b \in \{T,G,C\}, p(o_i^{(n,l)} = b) &= \sum_{b' \in \{A,T,G,C\}} p(o_i^{(n,l)} = b | o_{z_i^{(n)}}^{(n-1,l)} = b') p(o_{z_i^{(n)}}^{(n-1,l)} = b') \\ &= (1-3p_m) p(o_{z_i^{(n)}}^{(n-1,l)} = b) + \sum_{b' \in \{A,T,G,C\} \setminus b} p_m p(o_{z_i^{(n)}}^{(n-1,l)} = b') \\ &= (1-3p_m) p(o_{z_i^{(n)}}^{(n-1,l)} = b) + p_m \sum_{b' \in \{A,T,G,C\} \setminus b} p(o_{z_i^{(n)}}^{(n-1,l)} = b') \\ &= (1-3p_m) p(o_{z_i^{(n)}}^{(n-1,l)} = b) + p_m (1 - p(o_{z_i^{(n)}}^{(n-1,l)} = b)) \\ &= p_m + (1-4p_m) p(o_{z_i^{(n)}}^{(n-1,l)} = b) \end{aligned} \tag{21}$$

Given that $p(o_i^{(0,l)} = A) = 1$ by the particular case we consider here, the following recurrence ensues:

$$\begin{aligned}
&\text{For } b \in \{T,G,C\}, p(o_i^{(0,l)} = b) = 0 \\
&\qquad p(o_i^{(1,l)} = b) = p_m + (1-4p_m)\cdot 0 = p_m \\
&\qquad p(o_i^{(n,l)} = b) = p_m + \sum_{h=0}^{n-1}(1-4p_m)^h = p_m\left[\frac{1-(1-4p_m)^n}{1-(1-4p_m)}\right] = .25\left[1-(1-4p_m)^n\right]
\end{aligned} \tag{22}$$

Applying these results back in eqn. (18), we obtain the inheritance probability:

$$\begin{aligned}
p(\xi_i^{(n,l)} = 1 | \xi_{z_i^{(n)}}^{(n-1,l)} = 1) &= \sum_{b\in T,G,C}(1-p_m)p(o_{z_i^{(n)}}^{(n-1,l)} = b) \\
&= \sum_{b\in T,G,C}(1-p_m)\cdot .25\left[1-(1-4p_m)^{n-1}\right] \\
&= 3\times(1-p_m)\cdot .25\left[1-(1-4p_m)^{n-1}\right] \\
&= .75\cdot(1-p_m)\cdot \underbrace{\left[1-(1-4p_m)^{n-1}\right]}_{f^{(n-1)}} \\
&:= \tilde{p}_{inh}^{(n)}
\end{aligned} \tag{23}$$

where we observe that the inheritance probability is only a function of the level in the tree. The non-dependency of this rate on location, node index i and nucleotide type is made explicit in the notation.

1.4 On expected substitution numbers

Observe:

$$\begin{aligned}
E[S_i^{(n)} | S_{z_i^{(n)}}^{(n-1)}] &= E[X_{i1}^{(n)} | S_{z_i^{(n)}}^{(n-1)}] + E[X_{i2}^{(n)} | S_{z_i^{(n)}}^{(n-1)}] \\
&= S_{z_i^{(n)}}^{(n-1)} \tilde{p}_{inh}^{(n)} + (L - S_{z_i^{(n)}}^{(n-1)})\cdot 3p_m \\
&= S_{z_i^{(n)}}^{(n-1)}\left[\tilde{p}_{inh}^{(n)} - 3p_m\right] + 3p_m\cdot L \\
\implies E[S_i^{(n)}] &= E[S_{z_i^{(n)}}^{(n-1)}]\cdot\left[\tilde{p}_{inh}^{(n)} - 3p_m\right] + 3p_m\cdot L
\end{aligned} \tag{24}$$

As before, we fix $i = 1$, and consider a single sequence of parent-child relationships represented in the binary tree: $S_1^{(0)} \rightarrow S_1^{(1)} \rightarrow S_1^{(2)} \ldots \rightarrow S_1^{(2^N)}$.

$$
\begin{aligned}
E[S_1^{(0)}] &= 0 \\
E[S_1^{(1)}] &= E[E[S_1^{(1)}|S_1^{(n-1)}]] \\
&= E[S_1^{(1)}|S_1^{(n-1)} = 0] \\
&= 3p_m \cdot L \\
E[S_1^{(2)}] &= E[E[S_1^{(2)}|S_1^{(1)}]] \\
&= E[S_1^{(1)}][\tilde{p}_{inh}^{(2)} - 3p_m] + 3p_m \cdot L \\
&= 3p_m \cdot L[\tilde{p}_{inh}^{(2)} - 3p_m] + 3p_m \cdot L \\
E[S_1^{(3)}] &= E[E[S_1^{(3)}|S_1^{(2)}]] \\
&= E[S_1^{(2)}][\tilde{p}_{inh}^{(3)} - 3p_m] + 3p_m \cdot L \\
&= 3p_m \cdot L \cdot \left[1 + \sum_{a=2}^{3} \prod_{b=a}^{3} \left[\tilde{p}_{inh}^{(b)} - 3p_m\right]\right] \\
&\ldots \\
E[S_1^{(n)}] &= E[E[S_1^{(n)}|S_1^{(n-2)}]] \\
&= 3p_m \cdot L \cdot \left[1 + \sum_{a=2}^{n} \prod_{b=a}^{n} \left(\tilde{p}_{inh}^{(b)} - 3p_m\right)\right] \\
&\ldots
\end{aligned}
\tag{25}
$$

which is the result indicated in eqn. (11). The result makes it apparent that the right hand side is independent of the child node $i = 1$ for which these calculations were made and is only a function of generation number. Therefore the expected number of substitutions is the same for all 2^n nodes at a level n, and applies to all sequences of parent-child relationships specified by the binary tree.

To show that $\lim_{n\to\infty} E[S_i^{(n)}]$ exists, first observe that it was derived above that $3p_m$ is the mutation probability for a native nucleotide to switch to a substituted state. So the model defines a natural constraint on p_m: $3p_m < 1$ or $p_m < \frac{1}{3}$. Consider the ratio of product terms:

$$
\begin{aligned}
\left|\frac{\prod_{b=a}^{n+1}(\tilde{p}_{inh}^{(b)} - 3p_m)}{\prod_{b=a}^{n}(\tilde{p}_{inh}^{(b)} - 3p_m)}\right| &= \left|\tilde{p}_{inh}^{(n+1)} - 3p_m\right| \\
&= \left|.75(1-p_m)(1-(1-4p_m)^{n+1}) - 3p_m\right| \\
&< 1 \text{ for } p_m \in \left(0, \frac{1}{3}\right)
\end{aligned}
\tag{26}
$$

So the series $\lim_{n\to\infty} \sum_{a=1}^{n} \prod_{b=a}^{n} \left(\tilde{p}_{inh}^{(b)} - 3p_m\right)$ is absolutely convergent. By the comparison lemma for convergent sequences, $\lim_{n\to\infty} \sum_{a=2}^{n} \prod_{b=a}^{n} \left(\tilde{p}_{inh}^{(b)} - 3p_m\right)$ exists as well. So $\lim_{n\to\infty} E[S_i^{(n)}] = \lim_{n\to\infty} 3p_m L \left[1 + \sum_{a=2}^{n} \prod_{b=a}^{n} \left(\tilde{p}_{inh}^{(b)} - 3p_m\right)\right]$ exists.

To derive the bounds in eqn. (12), observe $\tilde{p}_{inh}^{(n)}$ is a monotonically increasing sequence in n, bounded above by

$.75(1-p_m)$. So $\left(\tilde{p}_{inh}^{(n)} - 3p_m\right) \geq \left(\tilde{p}_{inh}^{(m)} - 3p_m\right)$ for all $n > m$. Furthermore, for $p_m \in (0, \frac{1}{3}]$, $|\tilde{p}_{inh}^{(n)} - 3p_m| < 1$ for all n. So:

$$\begin{aligned}
3p_mL\left[1+\sum_{a=2}^{n}\prod_{b=a}^{n}\left(\tilde{p}_{inh}^{(b)} - 3p_m\right)\right] &\leq 3p_mL\left[1+\sum_{a=2}^{n}\prod_{b=a}^{n}\left(\tilde{p}_{inh}^{(n)} - 3p_m\right)\right] \\
&= 3p_mL\left[1+\sum_{a=2}^{n}\left(\tilde{p}_{inh}^{(n)} - 3p_m\right)^{n-a+1}\right] \\
&= 3p_mL\left[\sum_{a=0}^{n-1}\left(\tilde{p}_{inh}^{(n)} - 3p_m\right)^{a}\right] \\
&= 3p_mL\left(\frac{1-\left(\tilde{p}_{inh}^{(n)} - 3p_m\right)^{n}}{1-\left(\tilde{p}_{inh}^{(n)} - 3p_m\right)}\right)
\end{aligned} \tag{27}$$

1.5 On the probability distribution over substitution numbers

Write the conditional probabilities in eqn. (10) equivalently as:

$$\begin{aligned}
S_1^{(0)} &= 0, \text{ number of substitutions at root node is 0} \\
p\left(S_i^{(n)} = s_c | S_{z_i^{(n)}}^{(n-1)} = s_p\right) &= \sum_{k=0}^{s_c} Binomial(k|s_p, \tilde{p}_{inh}^{(n)}) \cdot Binomial(s_c - k|L - s_p, 3p_m) := \Gamma(s_c, s_p; n)
\end{aligned} \tag{28}$$

With eqn. (28), numerically compute an $(L+1)\times(L+1)$ conditional probability matrix Γ for every amplification cycle n. Similarly, conditioning on the substitutions of the parent node, the child node's substitutions are conditionally independent of the rest of the nodes on the tree. So for any non-root node i at any level n, write:

$$\begin{aligned}
p\left(S_i^{(n)} = s_c\right) &= \sum_{s_p=0}^{L} p\left(S_i^{(n)} = s_c | S_{z_i^{(n)}}^{(n-1)} = s_p\right) \cdot p\left(S_{z_i^{(n)}}^{(n-1)} = s_p\right) \\
&= \sum_{s_p=0}^{L} \Gamma(s_c, s_p; n) \cdot p\left(S_{z_i^{(n)}}^{(n-1)} = s_p\right)
\end{aligned} \tag{29}$$

With eqn. (29), numerically compute $p(S_i^{(n)})$ for any level n.

To show that $p(S_i^{(n)}) = p(S_j^{(n)})$ for all $j = 1\ldots 2^n$, and for all n, a proof by induction follows. The statement holds for $n = 1$.

$$\begin{aligned}
p(S_1^{(0)}) &= 0 \text{ by construction.} \\
p(S_1^{(1)} = s_c) &= \sum_{s_p=0}^{L} p(S_1^{(1)} = s_c | S_1^{(0)} = s_p) p(S_1^{(0)} = s_p) \\
&= p(S_1^{(1)} = s_c | S_1^{(0)} = 0) \\
&= \Gamma(0, s_p; 1) \\
&= p(S_2^{(1)} = s_c | S_1^{(0)} = 0) \\
&= \sum_{s_p=0}^{L} p(S_2^{(1)} = s_c | S_1^{(0)} = s_p) p(S_1^{(0)} = s_p) \\
&= p(S_2^{(1)} = s_c)
\end{aligned} \tag{30}$$

Assuming true for level n, it is shown that the statement holds for level $n+1$.

$$
\begin{aligned}
p(S_1^{(n+1)} = s_c) &= \sum_{s_p=0}^{L} p(S_1^{(n+1)} = s_c | S^{(n)}_{z_1^{(n+1)}} = s_p) p(S^{(n)}_{z_1^{(n+1)}} = s_p) \\
&= \sum_{s_p=0}^{L} \Gamma(s_c, s_p; n+1) p(S^{(n)}_{z_1^{(n+1)}} = s_p) \\
&= \sum_{s_p=0}^{L} \Gamma(s_c, s_p; n+1) p(S^{(n)}_{z_j^{(n+1)}} = s_p) \;\; \forall\, j = 2 \dots 2^{n+1} \\
&= \sum_{s_p=0}^{L} p(S_j^{(n+1)} = s_c | S^{(n)}_{z_j^{(n+1)}} = s_p) p(S^{(n)}_{z_j^{(n+1)}} = s_p) \\
&= p(S_j^{(n+1)} = s_c)
\end{aligned} \tag{31}
$$

1.6 $\psi(s|r)$ computation

For each sequencing read i, its position-specific Phred scores $q_i(k)$, over positions $k = 1 \dots L$ specify the conditional probability that the observed nucleotide at the position k is an error through the transformation $10^{-q_i(k)/10}$. Following the approximation noted in the subsection "Chaining amplification and sequencing", we calculate the average error rate for each read, $\bar{\lambda}_i = L \cdot \frac{1}{L} \sum_{k=1}^{L} 10^{-q_i(k)/10}$ and denote by $\hat{f}_\lambda$, their empirical density across reads. We can then numerically compute:

$$
\psi(S = s | r = 0) := \int_t p(s|t) \hat{f}_\lambda(t) dt \tag{32}
$$

where $p(s|t) = Poisson(t)$. Numerical calculations of other quantities like $q_\psi(\delta)$ follow in a straightforward fashion.

Calculation of $\psi(S|r)$ for all $r > 0$ follow in exactly the same fashion as eqn. (10) where the Binomials are replaced by the corresponding Poissons.

For computational tractability, we do these calculations over a random subset of $10,000$ sequencing reads.

References

[1] Moore, G. L. & Maranas, C. D. Modeling DNA Mutation and Recombination for Directed Evolution Experiments. *Journal of Theoretical Biology* **205**, 483–503 (2000). URL `http://www.sciencedirect.com/science/article/pii/S0022519300920824`.

[2] Pritchard, L., Corne, D., Kell, D., Rowland, J. & Winson, M. A general model of error-prone PCR. *Journal of Theoretical Biology* **234**, 497–509 (2005). URL `http://www.sciencedirect.com/science/article/pii/S0022519304006071`.

[3] Serfling, R. J. A General Poisson Approximation Theorem. *The Annals of Probability* **3**, 726–731 (1975). URL `https://www.jstor.org/stable/2959336`. Publisher: Institute of Mathematical Statistics.

[4] Robinson, M. D. & Oshlack, A. A scaling normalization method for differential expression analysis of RNA-seq data. *Genome biology* **11**, 1–9 (2010). Publisher: BioMed Central.

[5] Kumar, M. S. *et al.* Analysis and correction of compositional bias in sparse sequencing count data. *BMC genomics* **19**, 1–23 (2018). Publisher: Springer.

[6] Yarza, P. *et al.* Uniting the classification of cultured and uncultured bacteria and archaea using 16S rRNA gene sequences. *Nature Reviews Microbiology* **12**, 635–645 (2014). URL `https://www.nature.com/articles/nrmicro3330`. Number: 9 Publisher: Nature Publishing Group.
