# Supplementary material for: Differential richness inference for 16S rRNA marker gene surveys
Source: Genome Biol. 2022 Aug 1;23:166. doi: 10.1186/s13059-022-02722-x (PMC9344657; doi:10.1186/s13059-022-02722-x)
Supplement: Supplementary file 2 — Additional file 2. Presents supplementary figures S1-S22. Related literature references are contained within the note. [file 13059_2022_2722_MOESM2_ESM.pdf]

Additional File 2: Supplementary Figures

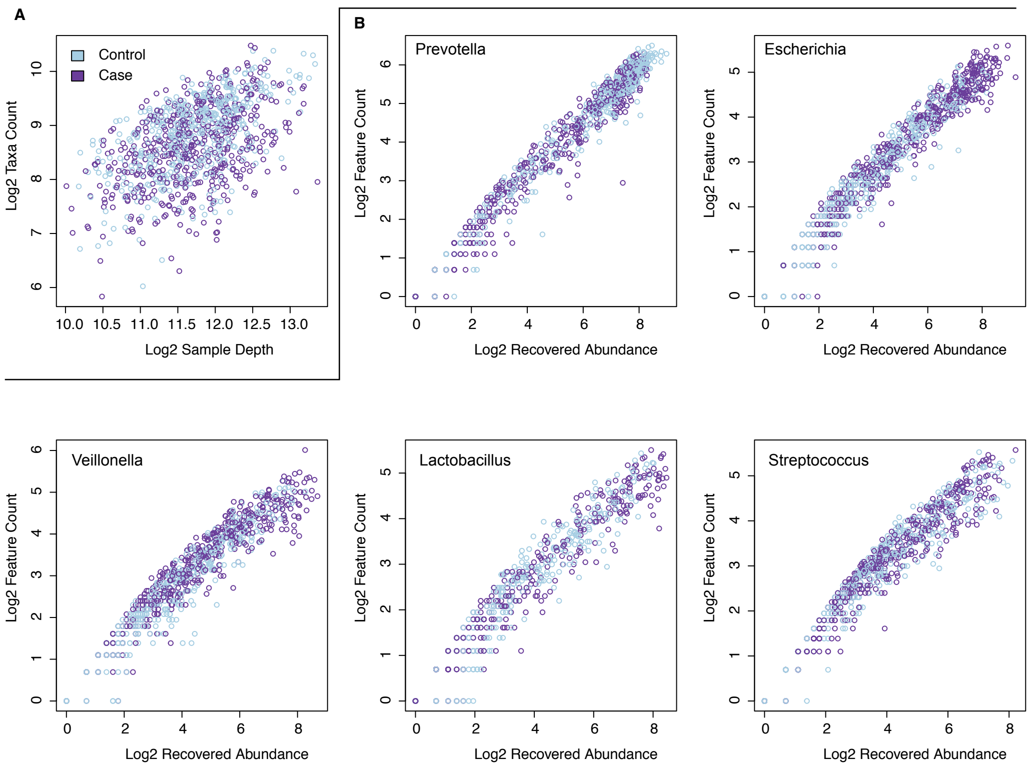

Fig. S1: Within-genus taxa accumulation data are concordant for several genera in 16S surveys. (A) Taxa accumulations with respect to sample-depth in the diarrheal microbiome dataset. (B) Genus-specific taxa accumulations with respect to the genus recovered abundance for a few genera in the same dataset.

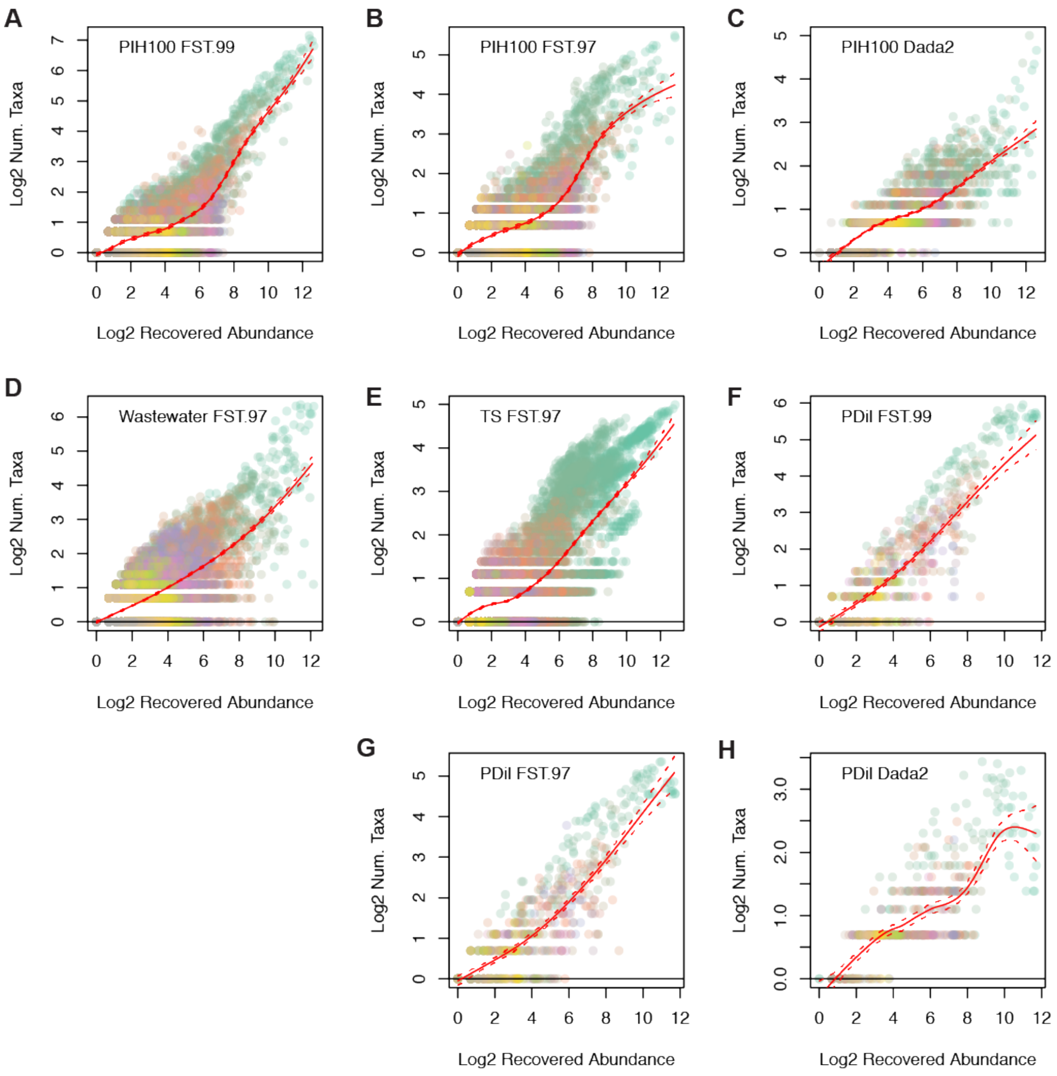

Fig. S2: Companion to the within-genus Table 1 in main text. Within-genus accumulations are overlaid across genus accumulations in each of the datasets. Smoothing spline $f_R(\cdot)$ within-genus accumulation trends for various datasets. Colors represent distinct genera within each sub-plot.

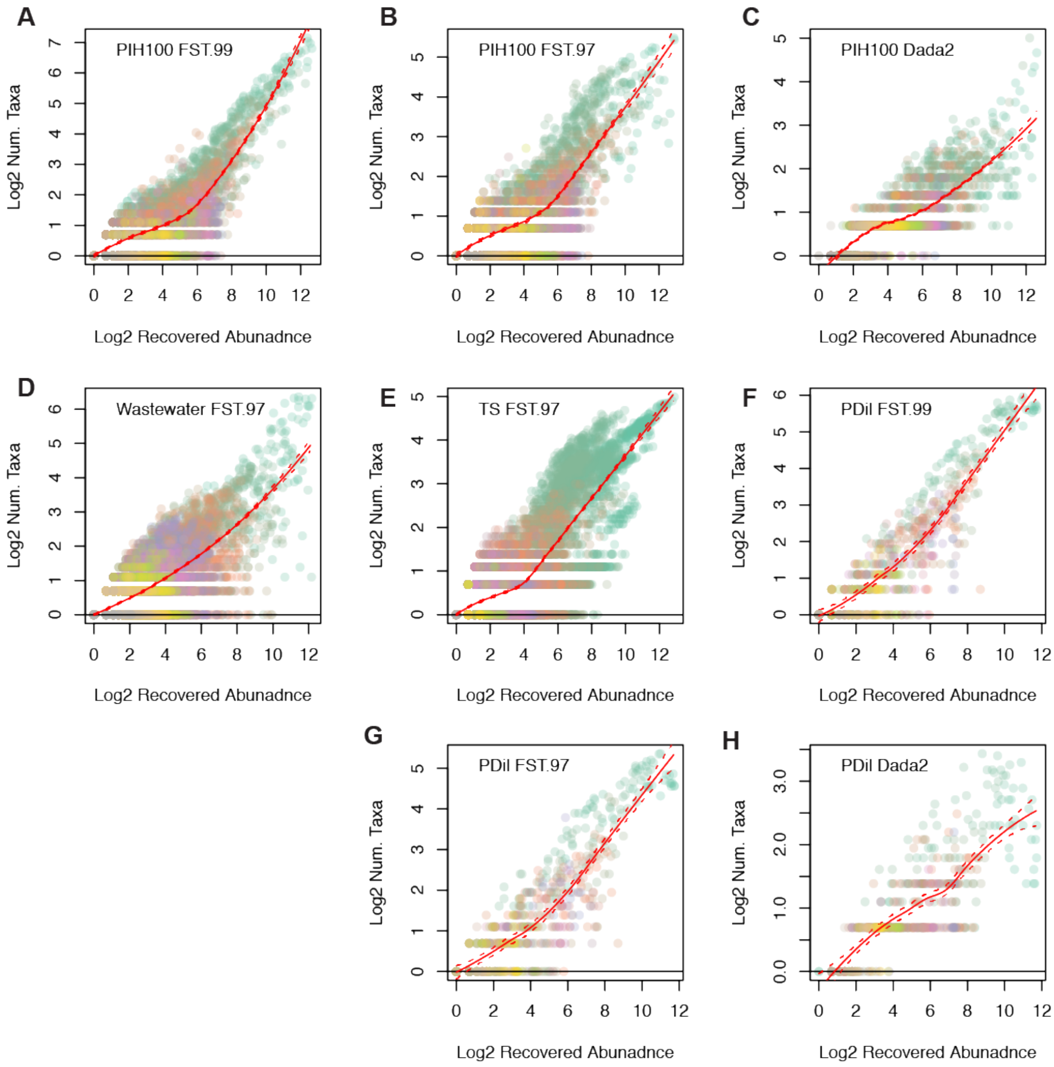

Fig. S3: Companion to within-genus Table 1 in main text. Within-genus accumulations are overlaid across genus accumulations in each of the datasets. Loess $f_R(\cdot)$ within-genus accumulation trends for various datasets. Colors represent distinct genera within each sub-plot.

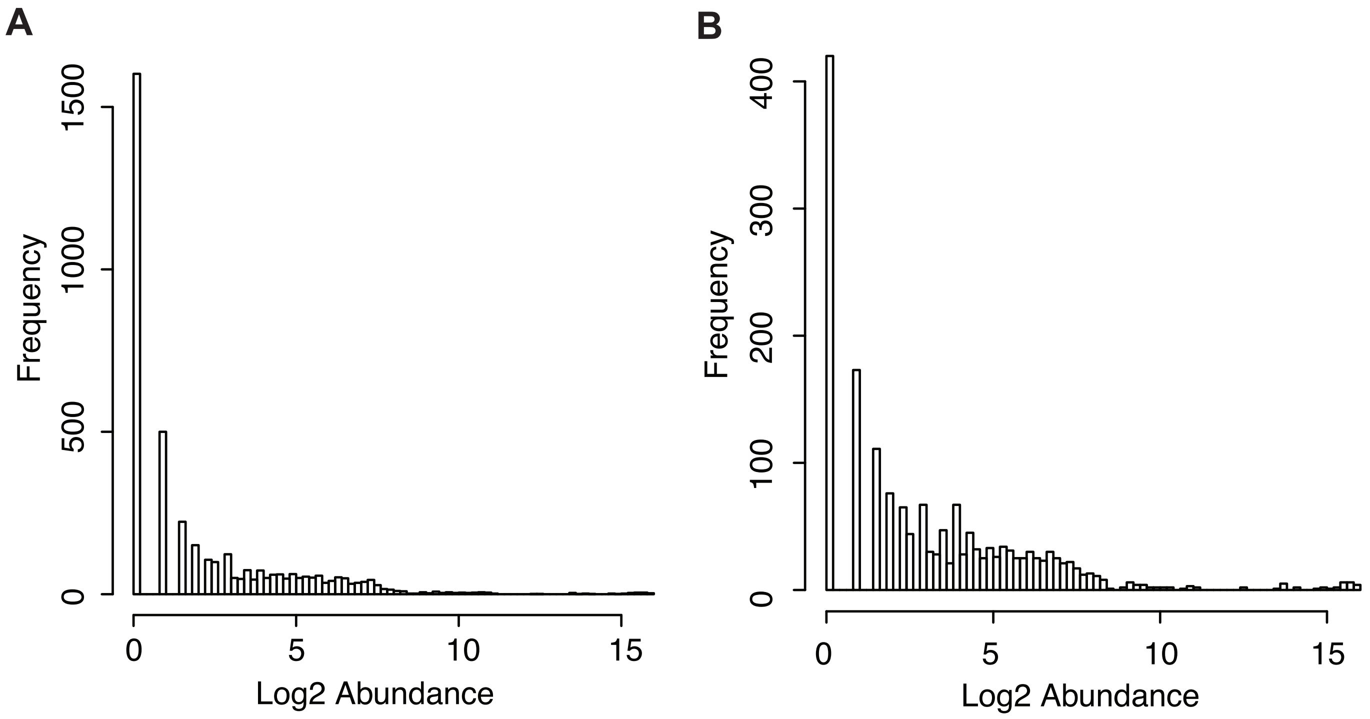

Fig. S4: Non-dominant Pseudomonas taxa discoveries from the dilution study preferentially contribute to the low frequency regime The Pseudomonas taxon with the largest dataset-wide aggregated count is taken as the dominant input sequence. Frequencies of frequencies of rest of the taxa are plotted for two FST clustering variants, (A) 99% and (B) 97%, including by including all samples from the dataset. The Pseudomonas genera is known to have ~ 4 16S rRNA genes per cell. Up to their sequence conservation, it is possible that larger counts are likely legitimate input sequences.

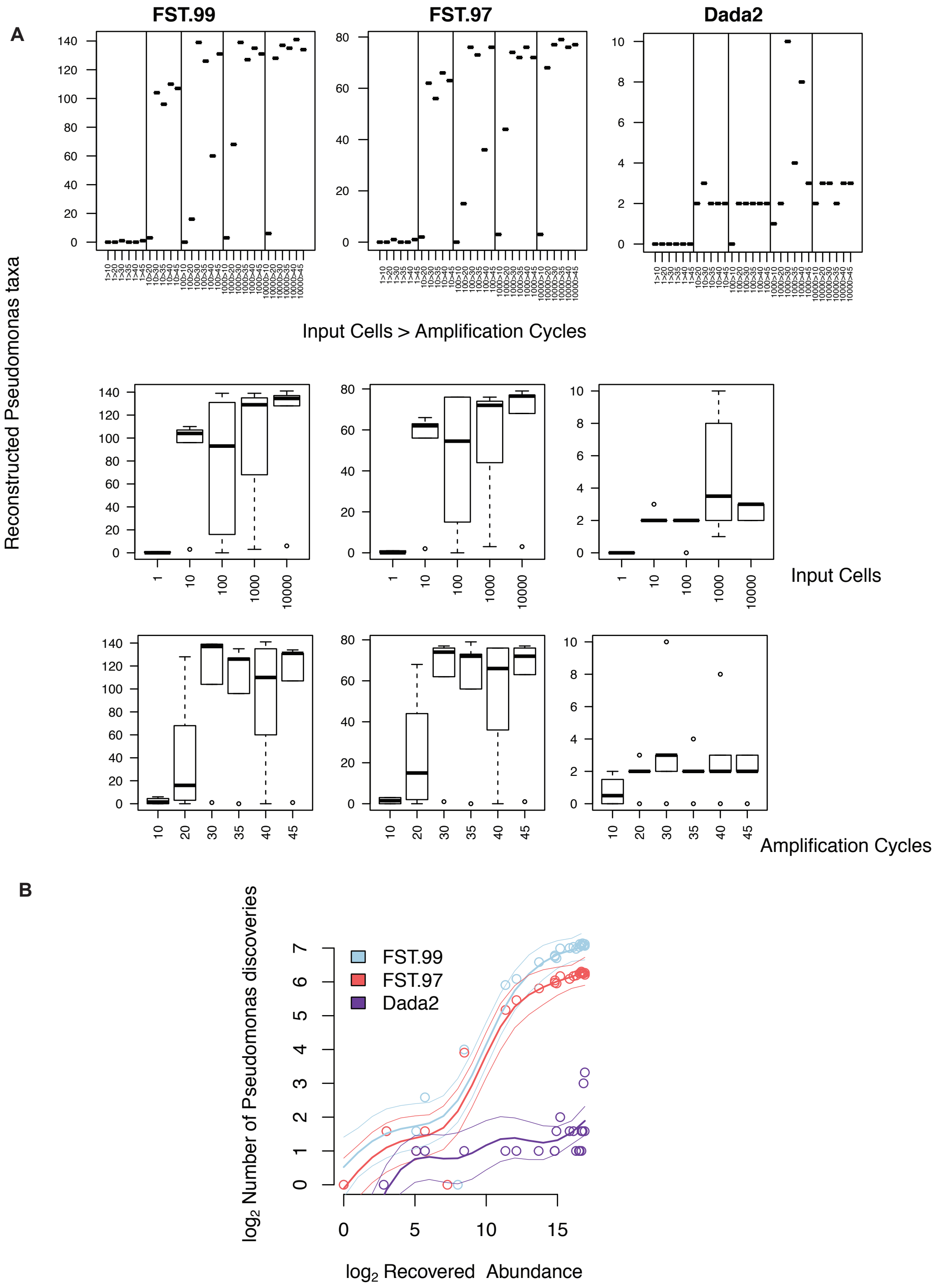

Fig. S5: False microbial discoveries accumulate along the recovered abundance axis As in main text Fig. 2, but only the taxa that were found to have a significant positive association with experimentally designed input cell numbers, as ascertained by a Poisson regression, are plotted. A caveat is that spurious discoveries have low frequencies, a regime where such tests have low detection power.

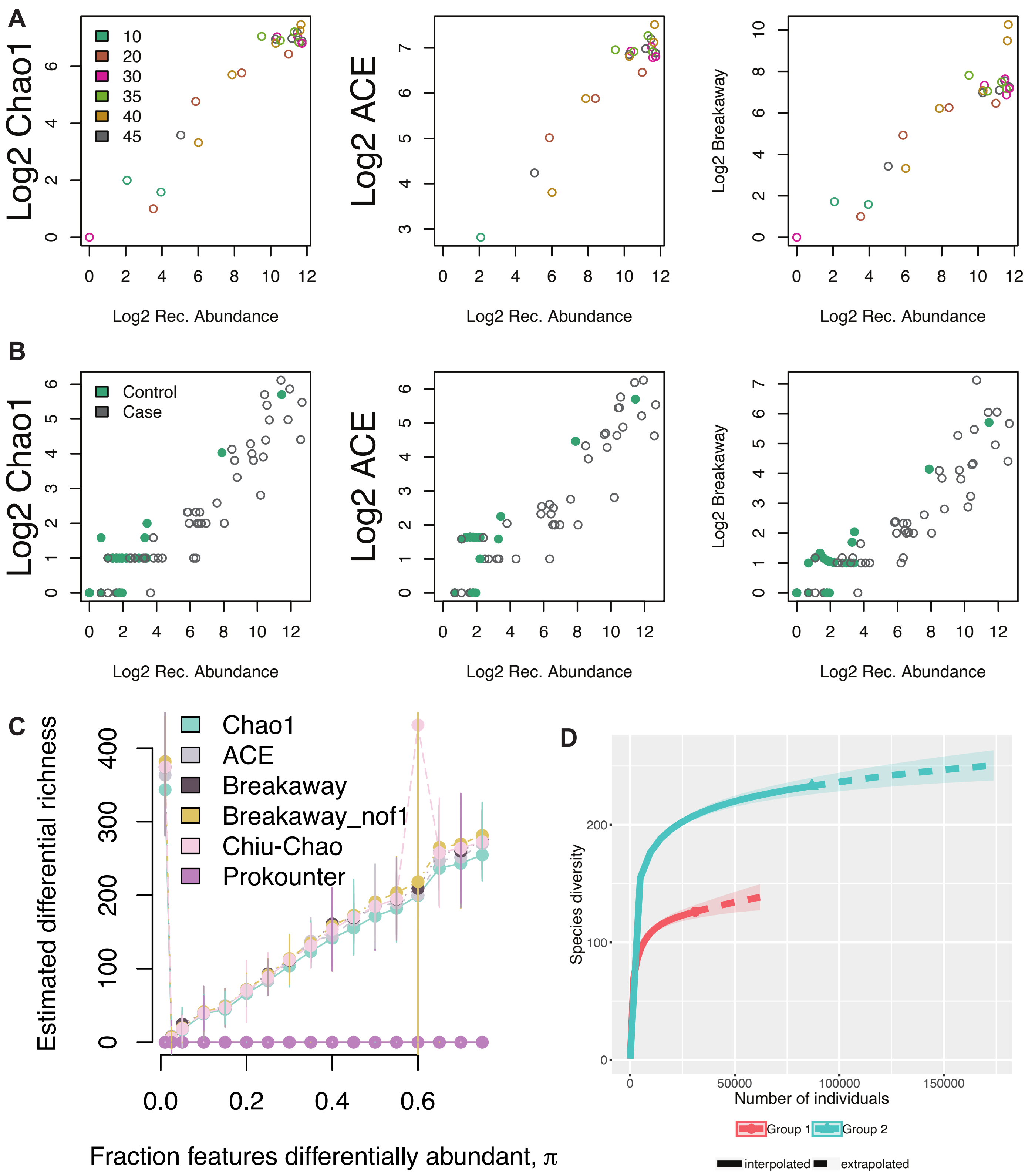

Fig. S6: Systematic bias in some common richness estimators. The estimated genus-specific richness grows as a function of genus-wide recovered abundance in groups of similar samples. (A) Sample-wise Pseudomonas genus richness estimates from the Pseudomonas experiment. Colors denote amplification cycles. (B) Sample-wise Paenibacillus genus richness estimates from PIH100 dataset. Colors indicate experimental groups. (C,D) Skewed relative abundance fold change distribution of detected genera across sample-groups lead to differential spurious taxa accumulations, resulting in confounded sample-wide richness inferences. Simulations are performed according to the procedure in additional file 5. Asymptotic richness estimators are noted in (C), and interpolated and extrapolated non-asymptotic rarefaction based richness inference for two simulated null samples are shown in (D).

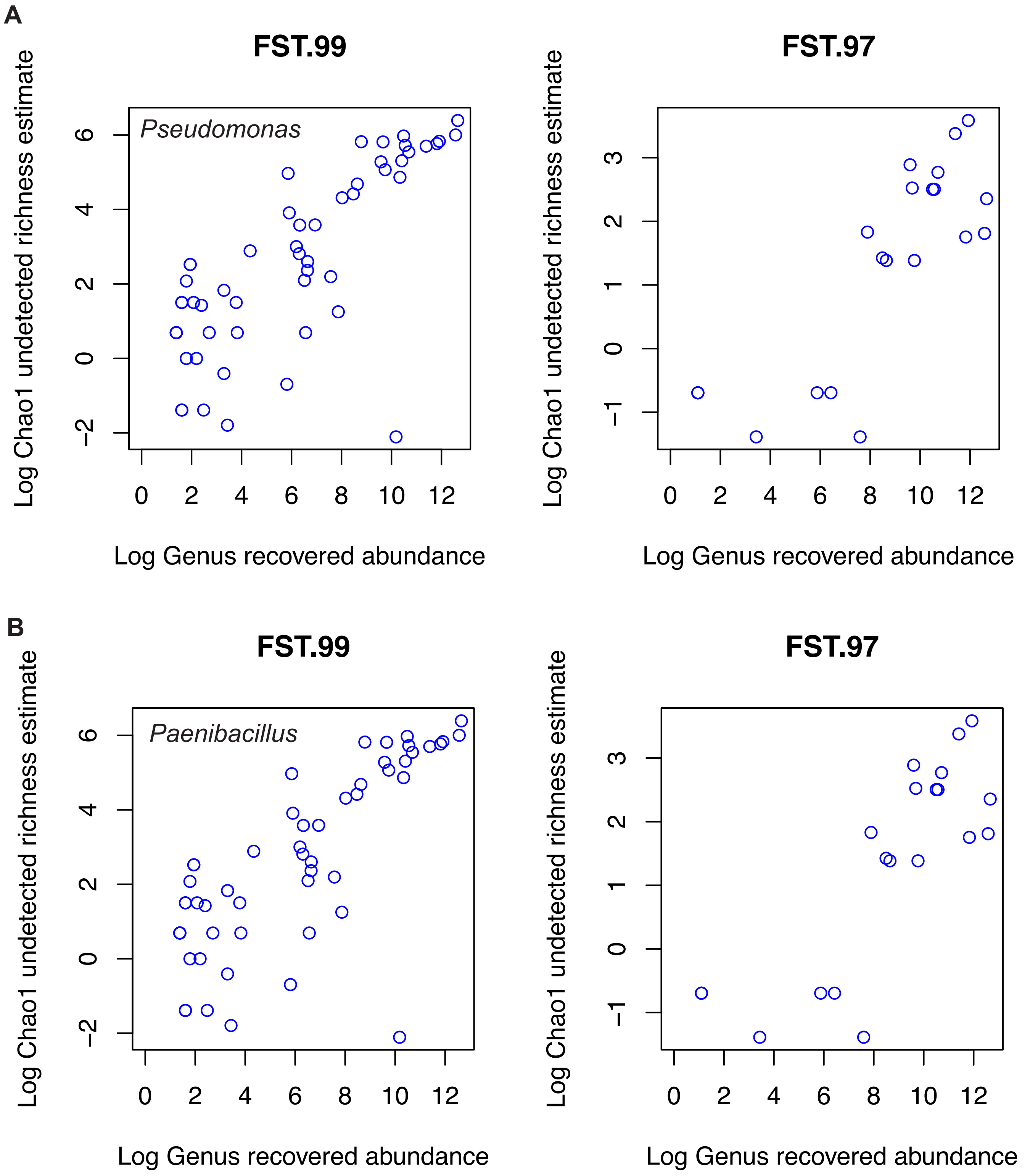

Fig. S7: Chao1 undetected richness estimates grow with genus recovered abundance The Chao1 undetected richness estimator for the Pseudomonas genus in the Pseudomonas dilution study (top row) and Paenibacillus genus in PIH100 survey (bottom row) for two different settings of sequence similarity thresholds. FST.99 and FST.97 correspond to sequence similarity thresholds of 99% and 97% respectively.

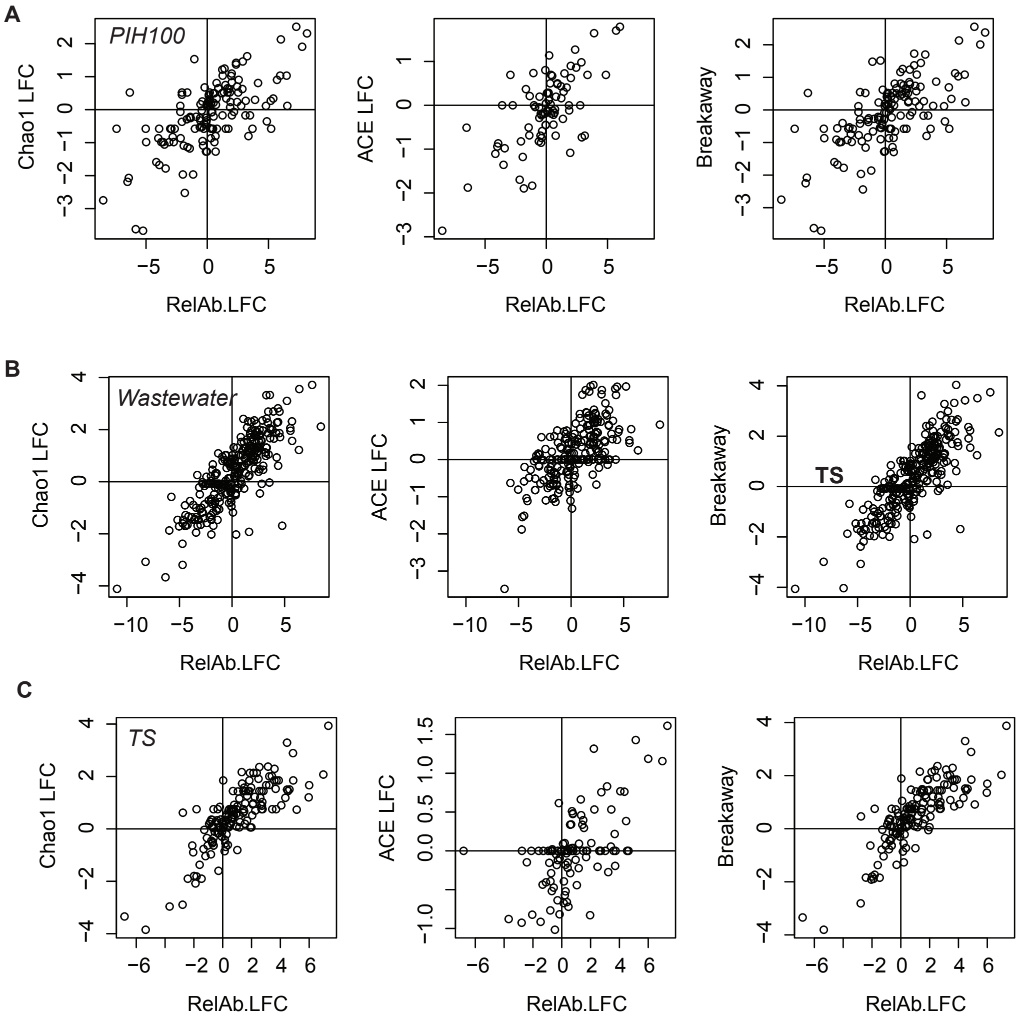

Fig. S8: Differential richness estimators are biased by differential abundance. Each row corresponds to a two-group comparison for a distinct 16S survey. (A) PIH100 (Case vs. control), (B) Wastewater (Effluent vs. Influent), (C) Long term time series (Post-infection vs. Pre-infection). In each case, the estimated log-fold changes in estimated richness by the noted estimators are plotted against the genus-wise relative abundance log-fold changes across sample groups. In each plot, those genera for which a DR estimate was not available due to estimation problems in respective methods, are not shown.

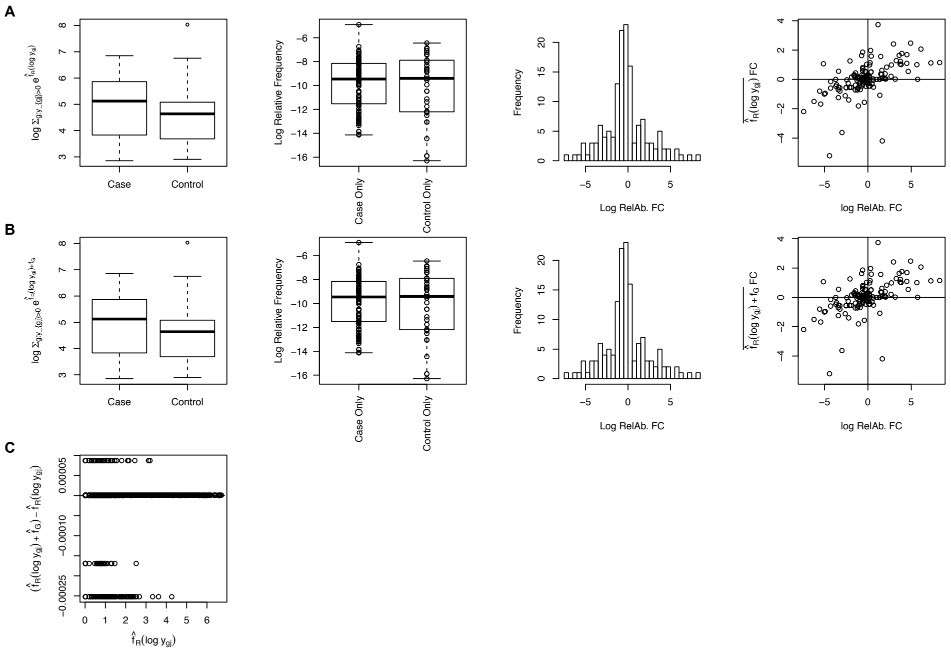

Fig. S9: A comparison of two technical component estimators for the PIH100 FST99 dataset. (A) without genus contributions, (B) with genus contributions. In both rows, the first column plots the technical predictor in each sample-group. Second column plots the relative abundances of genuses occurring in one of the sample-groups only. The third column plots the histogram of genus-wise case vs. control relative abundance fold changes. The fourth column plots the fold change in the technical predictor against the respective genera's relative abundances. (C) plots the two estimators.

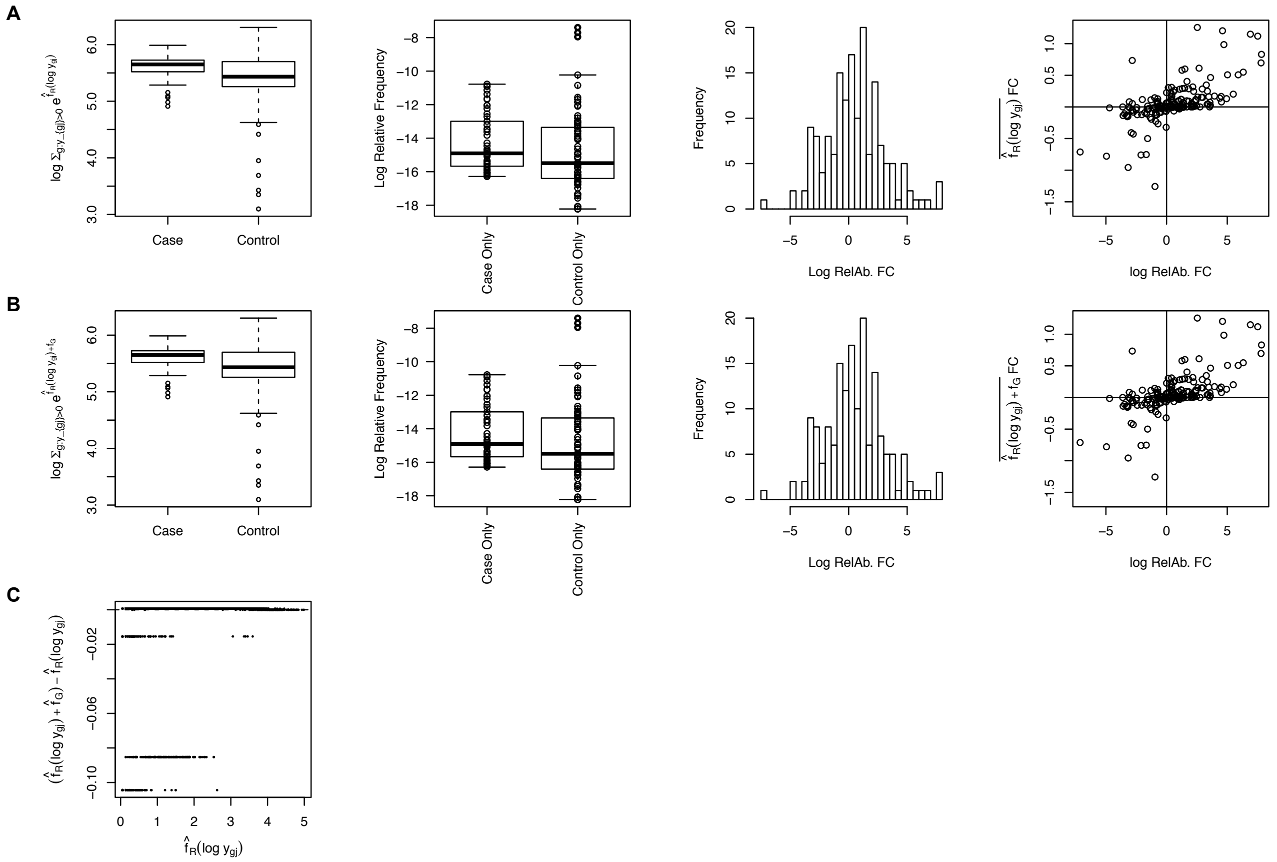

Fig. S10: A comparison of two technical component estimators for the TS FST97 dataset. Case and control refer to post-infection and pre-infection samples respectively. (A) without genus contributions, (B) with genus contributions. In both rows, the first column plots the technical predictor in each sample-group. Second column plots the relative abundances of genuses occurring in one of the sample-groups only. The third column plots the histogram of genus-wise case vs. control relative abundance fold changes. The fourth column plots the fold change in the technical predictor against the respective genera's relative abundances. (C) plots the two estimators.

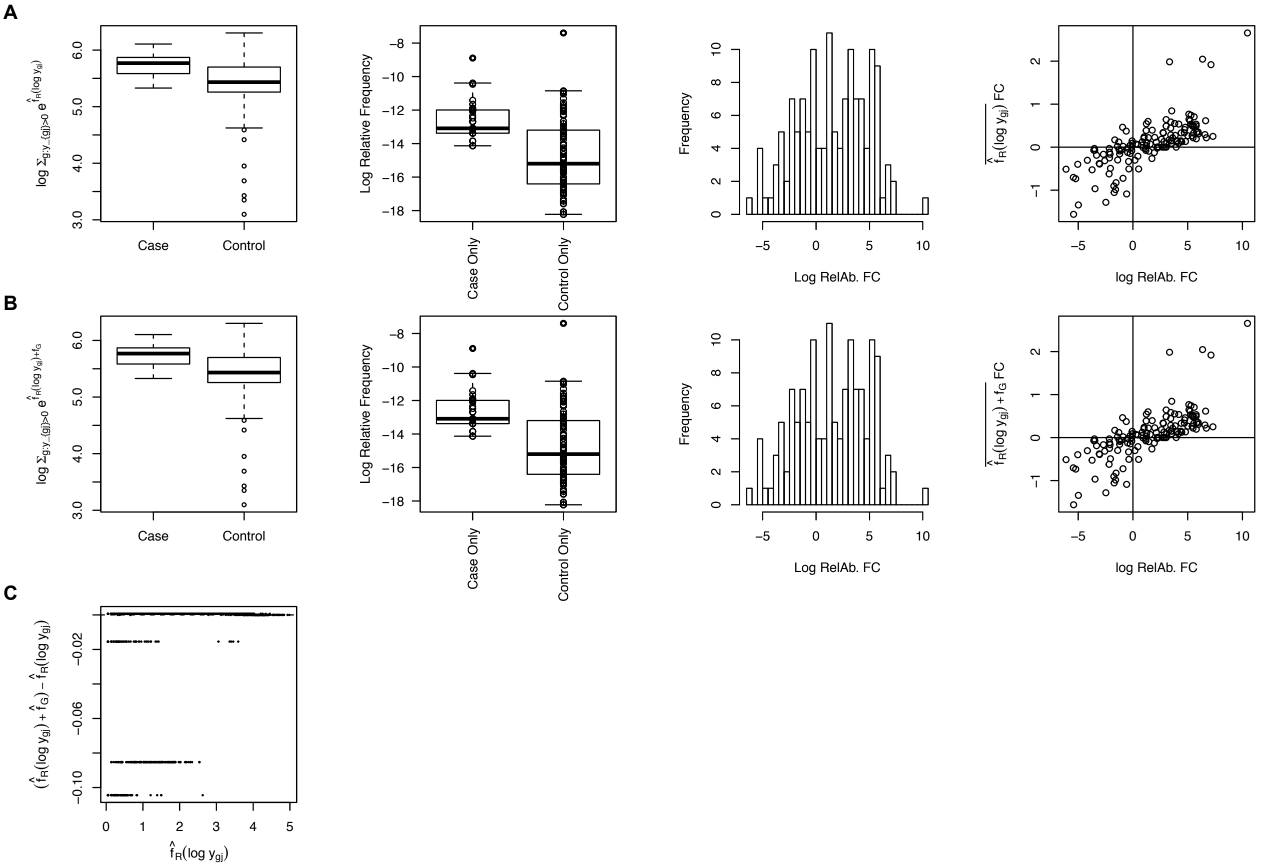

Fig. S11: A comparison of two technical component estimators for the TS FST97 dataset. Case and control refer to infection and pre-infection samples respectively. (A) without genus contributions, (B) with genus contributions. In both rows, the first column plots the technical predictor in each sample-group. Second column plots the relative abundances of genuses occurring in one of the sample-groups only. The third column plots the histogram of genus-wise case vs. control relative abundance fold changes. The fourth column plots the fold change in the technical predictor against the respective genera's relative abundances. (C) plots the two estimators.

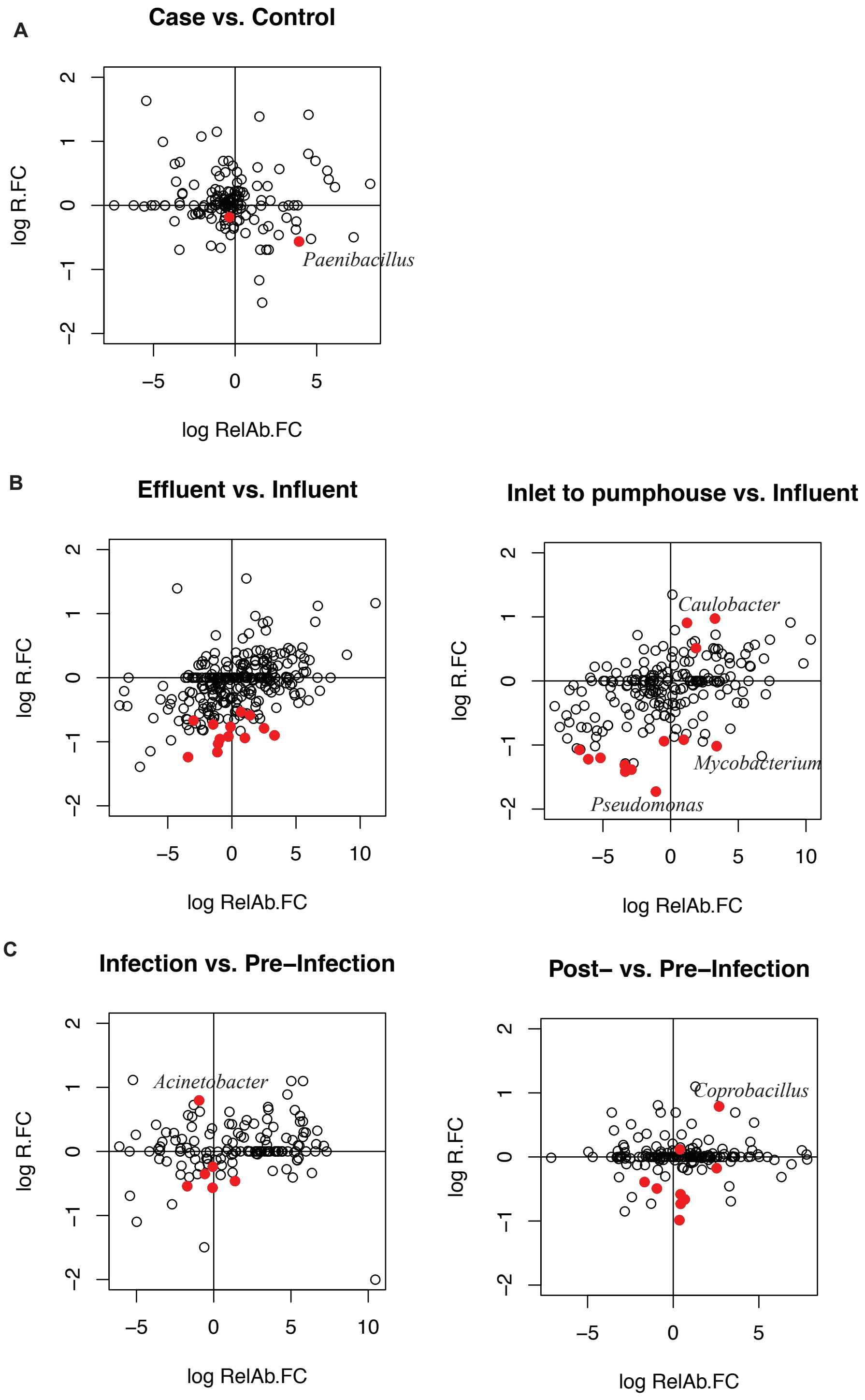

Fig. S12: Genus-specific differential richness inferences from Prokounter. Each row corresponds to a two-group comparison for a distinct 16S survey. Compare to supplementary Fig. S8. (A) PIH100 (Case vs. control), (B) Wastewater (Effluent vs. Influent), and (Inlet to pumphouse vs Influent), (C) Long term time series (Infection vs. Pre-infection, and Post-infection vs. Pre-infection). In each case, the estimated log-fold changes in estimated richness by the noted estimators are plotted against the genus-wise relative abundance log-fold changes across sample groups. Significant richness differences are colored red.

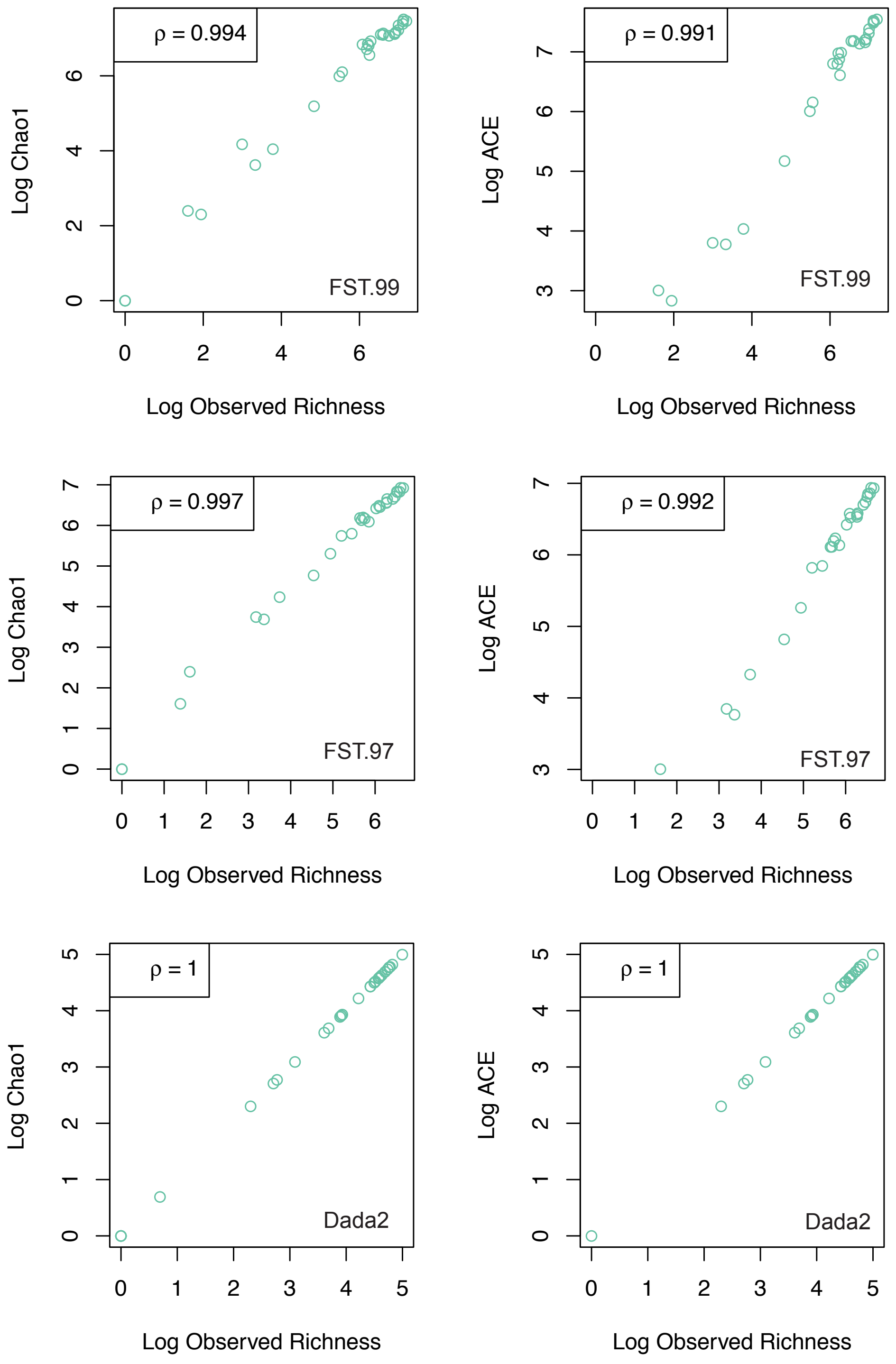

Fig. S13: Sample-wide richness estimates track observed sample-wide richness in the Pseudomonas dilution study, for various clustering methods mentioned. ρ refers to Pearson correlation.

.

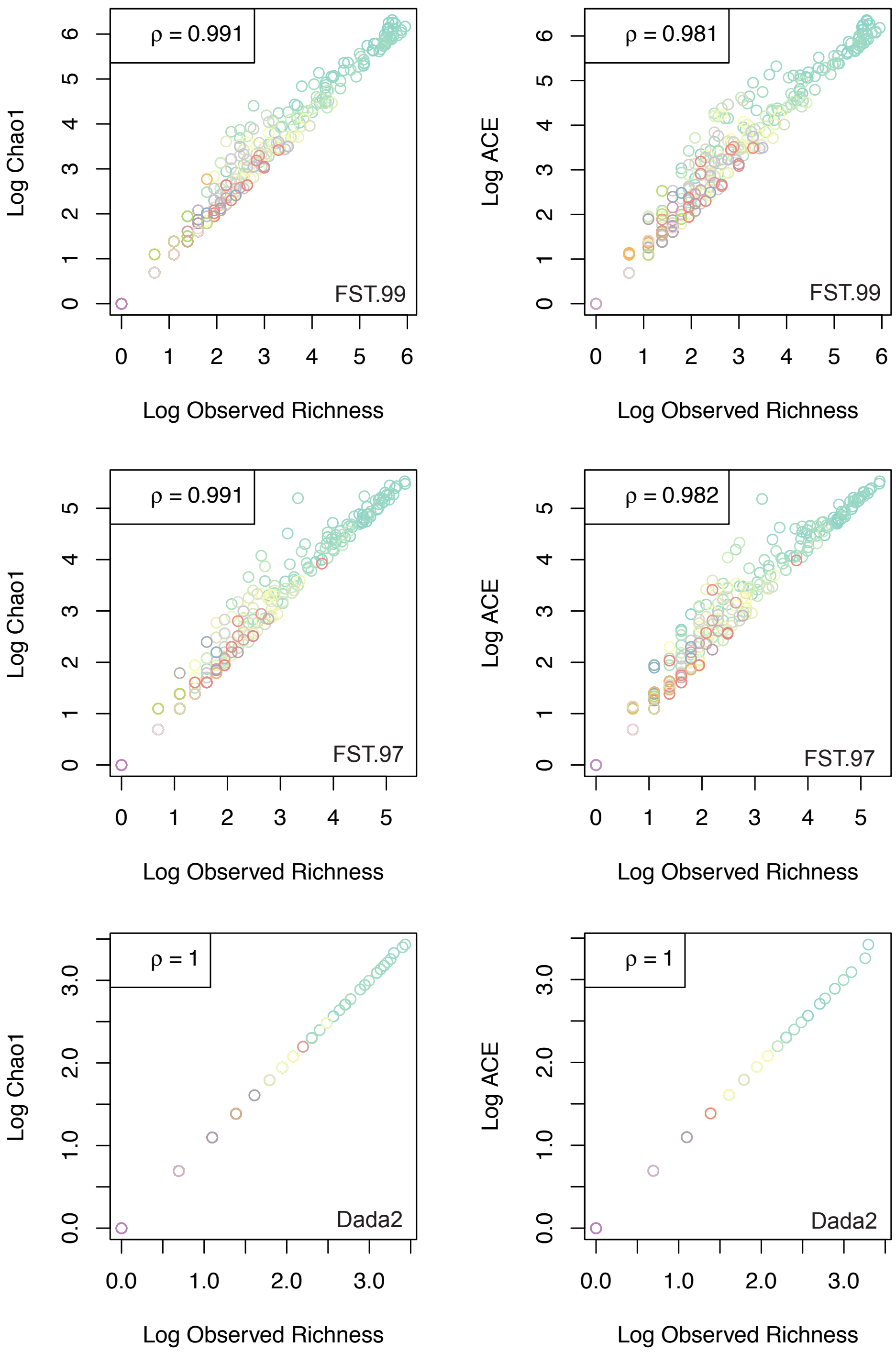

Fig. S14: Genus-specific richness estimates track genus-specific observed richness values in the Pseudomonas dilution study.

.

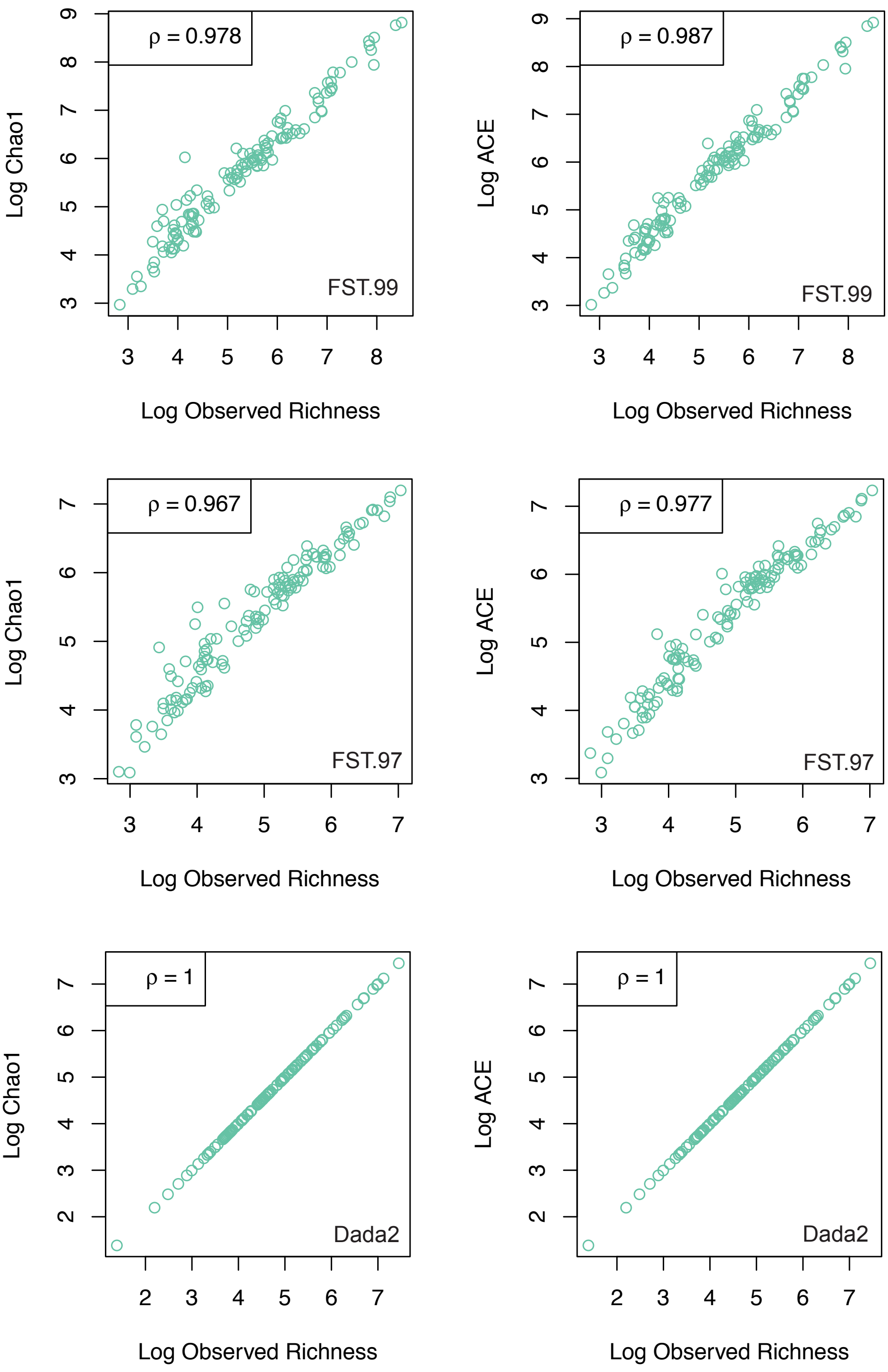

Fig. S15: Sample-wide richness estimates track observed sample-wide richness in the PIH100 study [1].

.

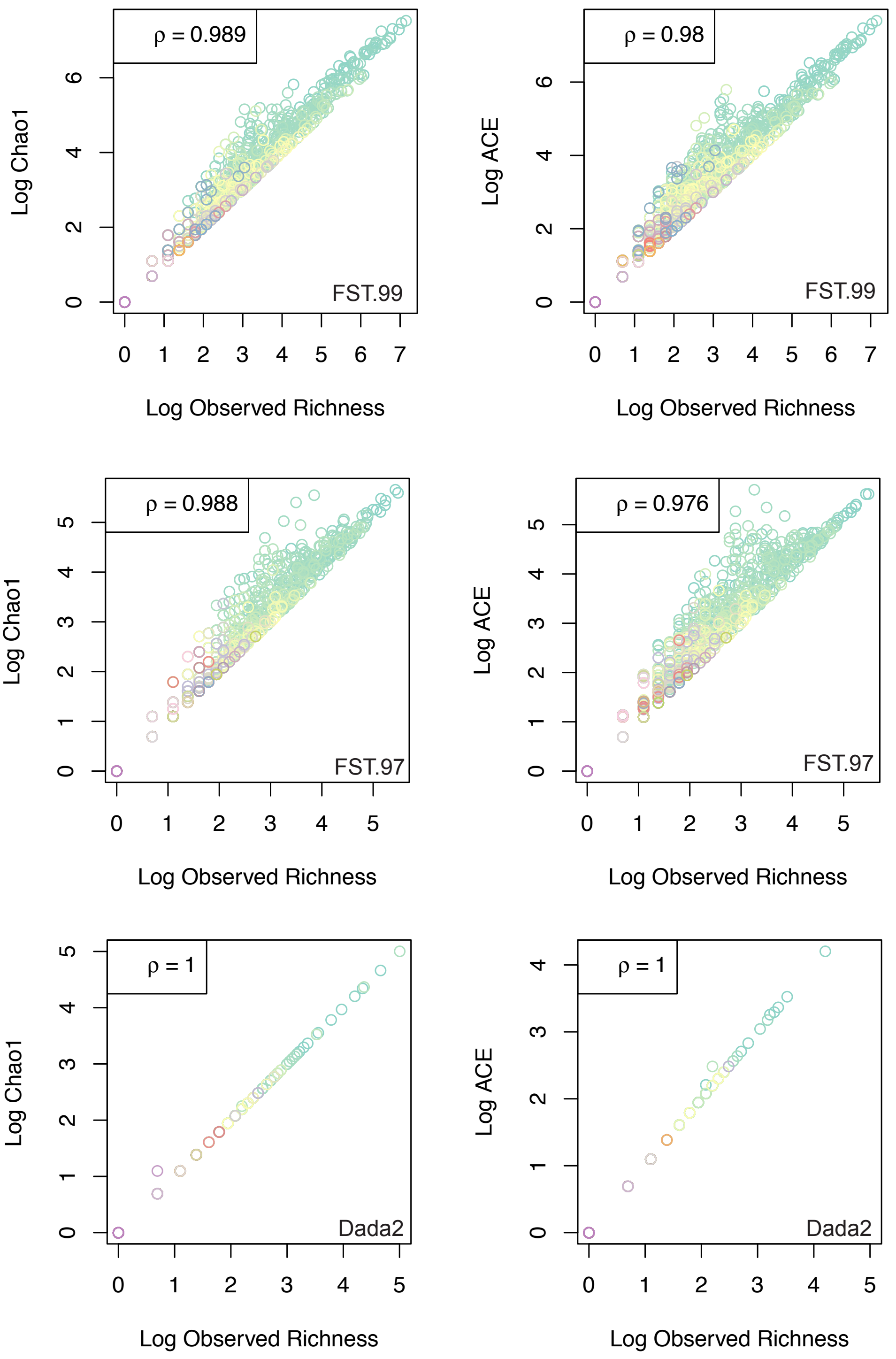

Fig. S16: Genus-specific richness estimates track genus-specific observed richness values in the PIH100 study [1].

.

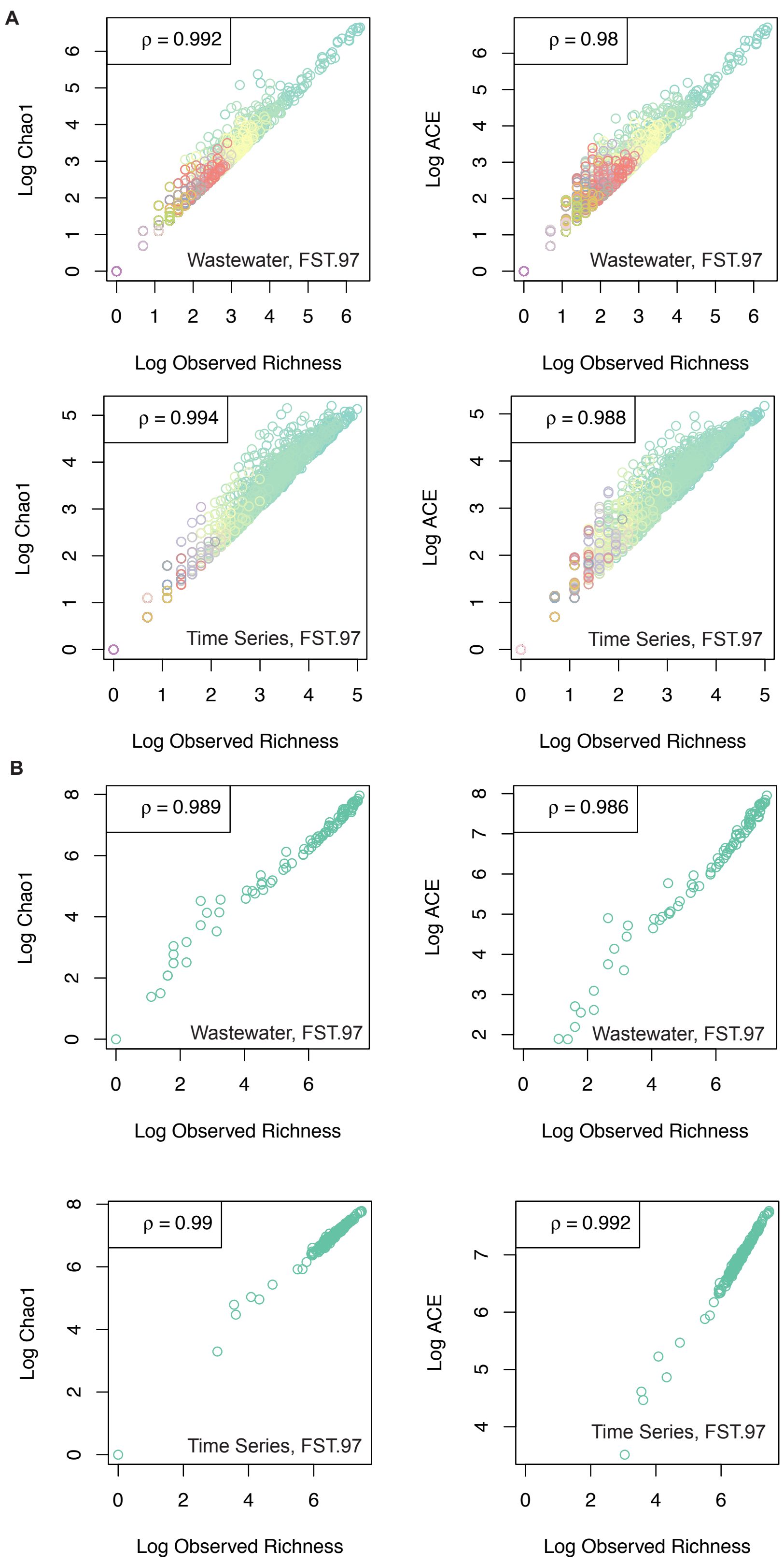

Fig. S17: (A) Genus-specific richness estimates track genus-specific observed richness values in the wastewater [2] and long-term time series study [3]. (B) Sample-wide richness estimates track observed sample-wide richness in Wastewater and long-term time series study.

.

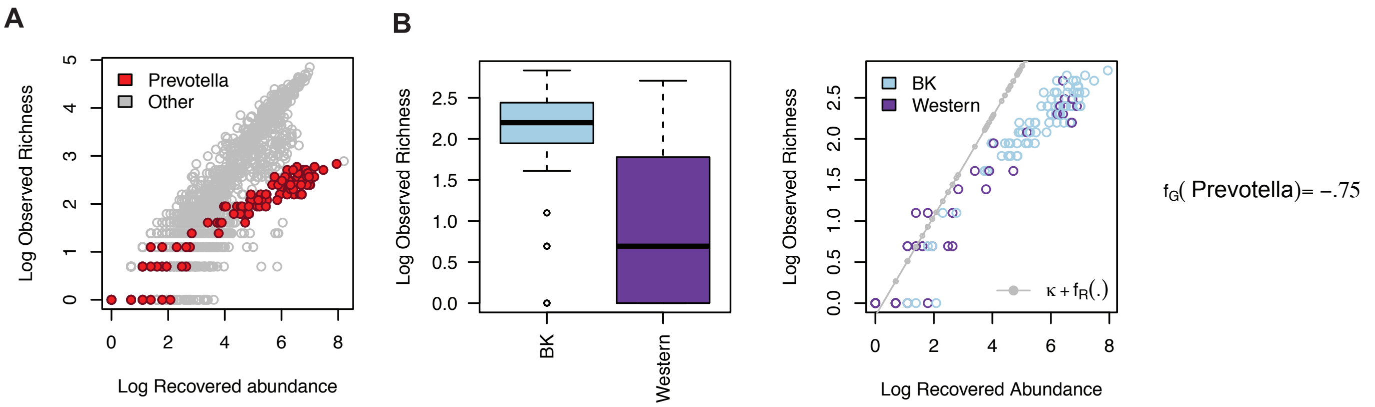

Fig. S18: (A) Relative to most of the genera, *Prevotella* has a systematically reduced accumulation trend through most of the recovered abundance range in the mouse diet study [4]. (B) This is captured by f_G in the `Prokounter` model. *Prevotella* also has reduced observed richness (number of taxa) in the "Western" group relative to "BK" control diet group. Unless $f_G()$ is incorporated, the abundance dependent technical correction indicated by $f_R()$ is too severe for *Prevotella*, leading one to entirely attribute the observed richness differences between groups to sampling abundance variation.

.

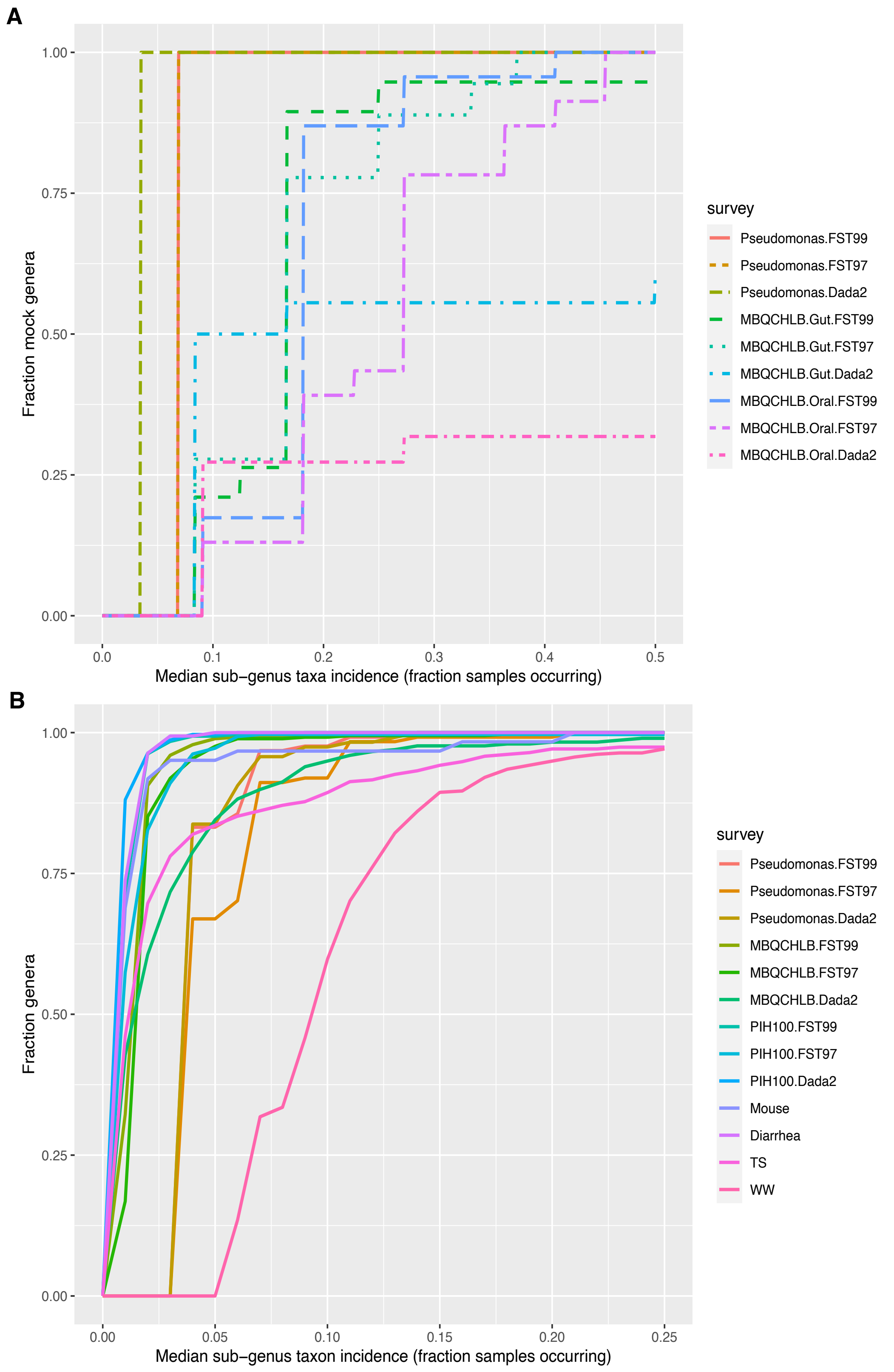

Fig. S19: Replicability of sub-genus taxa in study samples. For each genus in a given study, its median sub-genus taxa incidence is calculated as the median of the fraction of the study samples in which the genus's taxa occur. This value is plotted along the x-axis. For each incidence value along the x-axis, the y-axis plots the cumulative fraction of genera with at most that medium sub-genus taxa incidence value. (A) restricts this analysis to mock genera in mock datasets only and (B) generalizes this to all represented genera in all datasets considered in this paper.

.

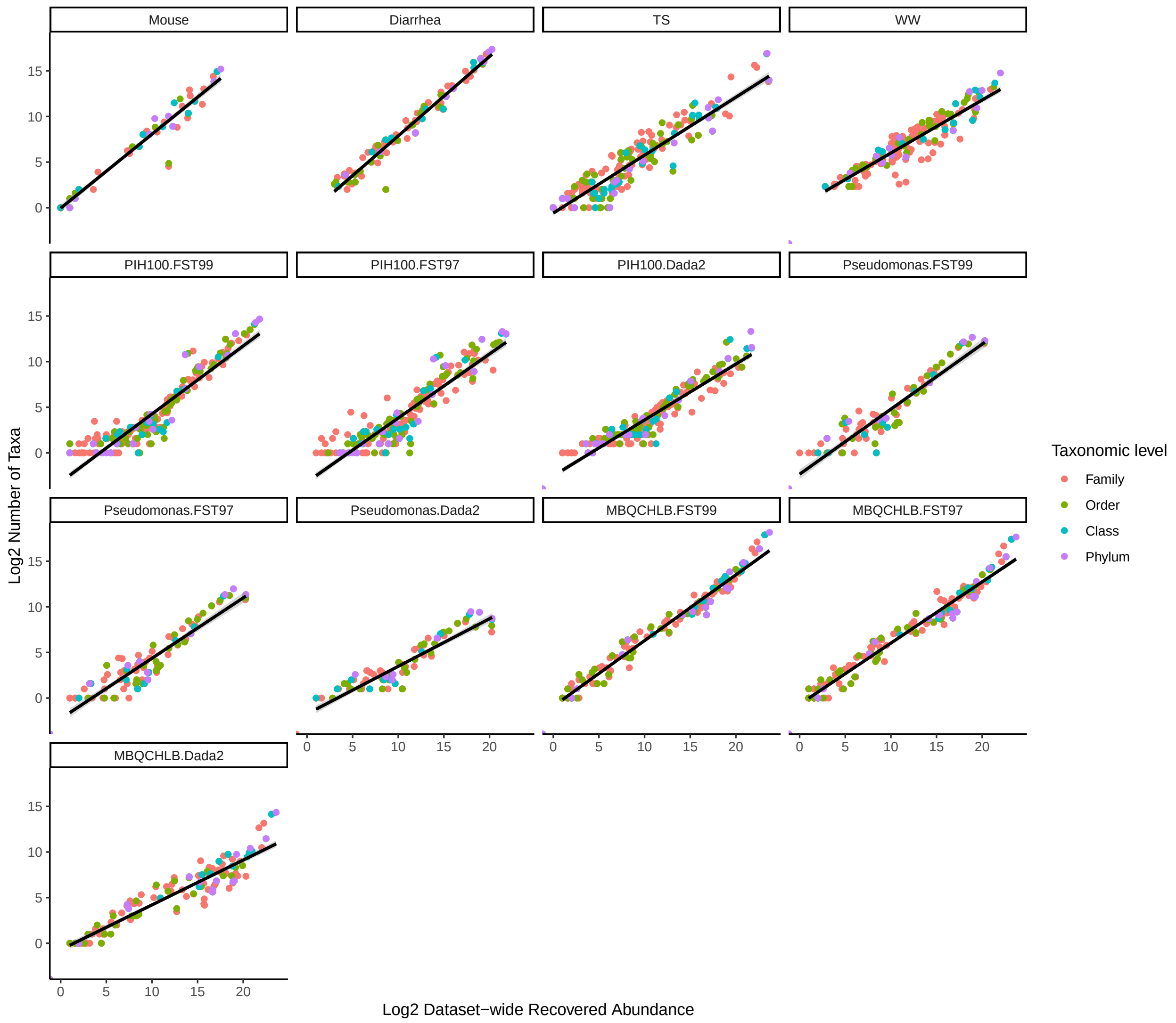

Fig. S20: Observed richness for any taxonomic level is strongly predicted by their recovered abundance alone and is not dependent on the taxonomic level. For each detected taxonomic level (i.e., family, order, class and phylum) in a given study (facets), its total dataset-wide recovered abundance is plotted along the x-axis. The y-axis plots the number of taxa for the taxonomic level. Black lines indicate the least squares fit obtained by fitting the logged number of taxa (y-axis) with the logged recovered abundances (x-axis).

.

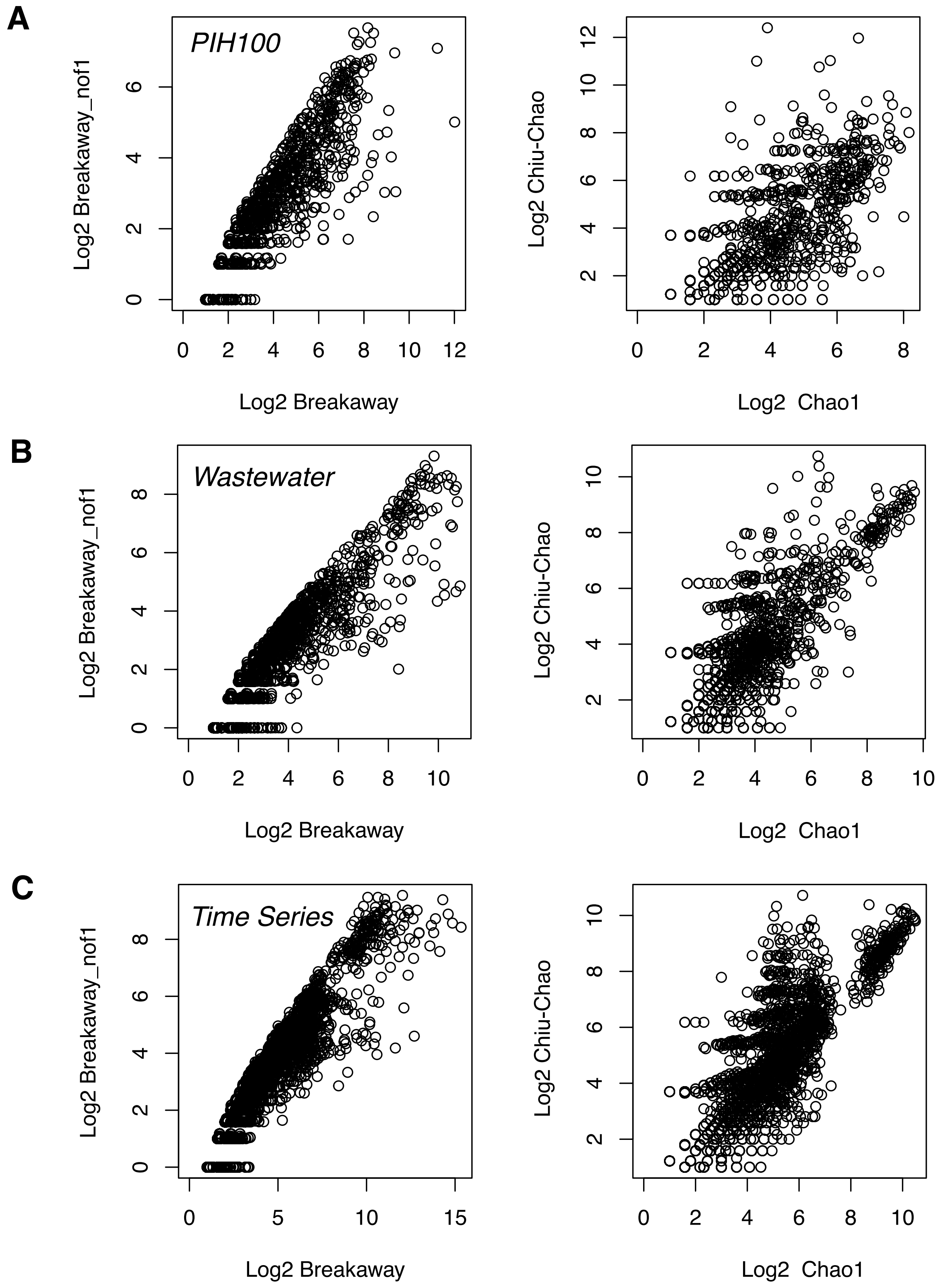

Fig. S21: Estimated richness from two sequencing-noise aware estimators (Breakaway_nof1 and Chiu-Chao) are plotted against their basic counterparts (Breakaway and Chao1) to demonstrate correlation. LFC is log-fold change. RelAb. relative abundance. Each row corresponds to a distinct study. (A) PIH100, (B) Waste-water treatment, and (C) time series.

.

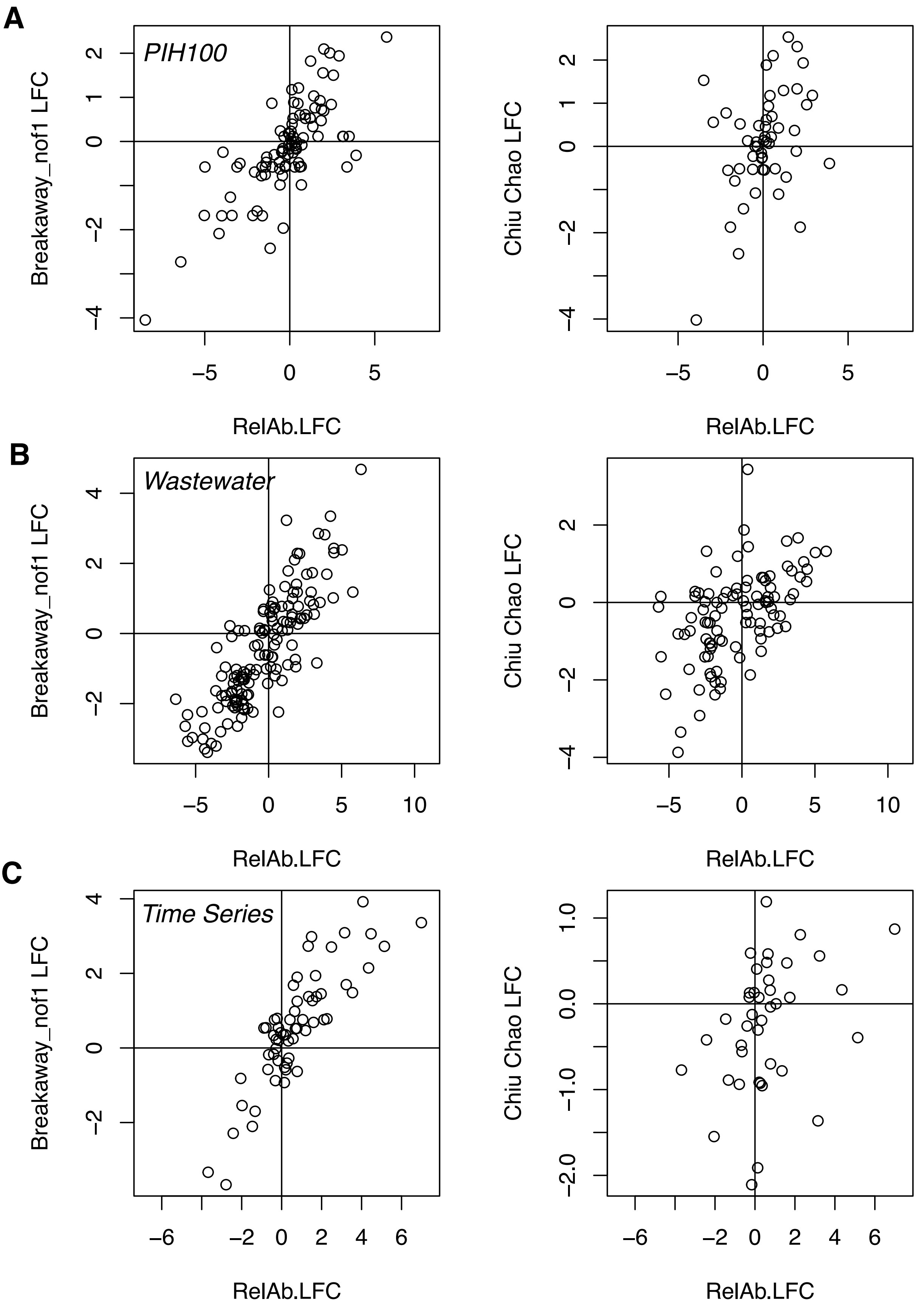

Fig. S22: Estimated genus-specific differential richness statistics from two sequencing-noise aware estimators (Breakaway_nof1 and Chiu-Chao) (y-axis) are plotted against genus-wise differential abundance statistics (x-axis) for the same data as in Fig. S8. The fewer number of points in each panel arises because of undefined numerical values. LFC, log-fold change. RelAb., relative abundance. Each row corresponds to a distinct study. (A) PIH100, (B) Waste-water treatment, and (C) time series.

References

[1] Paulson, J. N. *et al.* Paenibacillus infection with frequent viral coinfection contributes to postinfectious hydrocephalus in Ugandan infants. *Science translational medicine* **12** (2020). Publisher: American Association for the Advancement of Science.

[2] Kulkarni, P. *et al.* Conventional wastewater treatment and reuse site practices modify bacterial community structure but do not eliminate some opportunistic pathogens in reclaimed water. *Science of The Total Environment* **639**, 1126–1137 (2018). URL `https://www.sciencedirect.com/science/article/pii/S004896971831828X`.

[3] David, L. A. *et al.* Host lifestyle affects human microbiota on daily timescales. *Genome biology* **15**, 1–15 (2014). Publisher: BioMed Central.

[4] Turnbaugh, P. J. *et al.* The effect of diet on the human gut microbiome: a metagenomic analysis in humanized gnotobiotic mice. *Science translational medicine* **1**, 6ra14–6ra14 (2009). Publisher: American Association for the Advancement of Science.
