# Supplementary material for: Differential richness inference for 16S rRNA marker gene surveys
Source: Genome Biol. 2022 Aug 1;23:166. doi: 10.1186/s13059-022-02722-x (PMC9344657; doi:10.1186/s13059-022-02722-x)
Supplement: Supplementary file 3 — Additional file 3. Presents supplementary tables S1-S3. Related literature references are contained within the note. [file 13059_2022_2722_MOESM3_ESM.pdf]

Additional File 3: Supplementary Tables

Dataset	Year	16S segment, Sequencing& Clustering	Number of detected genera	Optimal number of clusters
Mouse [1, 2]	2009	V2, 454, FST.97	60	2
Diarrhea [3]	2014	V12, 454, FST.99	163	2
Time series [4]	2014	V4, GAIIx, FST.97	309	3
Wastewater [5]	2018	V34, MiSeq, FST.99	412	4
MBQC-HLB$^{(97)}$ [6]	2017	V4, MiSeq, FST.97	370	3
MBQC-HLB$^{(99)}$ [6]	2017	V4, MiSeq, FST.99	373	3
MBQC-HLB$^{(D)}$ [6]	2017	V4, MiSeq, Dada2	298	2
PIH100$^{(97)}$ [7]	2020	V12, MiSeq, FST.97	288	6
PIH100$^{(99)}$ [7]	2020	V12, MiSeq, FST.97	318	5
PIH100$^{(D)}$ [7]	2020	V12, MiSeq, Dada2	288	6
Pseudomonas$^{(97)}$	2021	V12, MiSeq, FST.97	124	2
Pseudomonas$^{(99)}$	2021	V12, MiSeq, FST.99	125	8
Pseudomonas$^{(D)}$	2021	V12, MiSeq, Dada2	117	5

Table S1: Within-genus taxa accumulation data organize into very few accumulation patterns. For each survey, its within-genus taxa accumulation data is organized into two columns as follows. The first column holds, the raw recovered abundance values for each genus for all samples, and the second column holds the corresponding number of observed taxa. The recovered abundance values were binned into 25 windows, with comparable number of observations (i.e., rows in the two column table) in each window. A data matrix was next generated with rows representing genus, columns representing each of the 25 windows, and each cell corresponding to the mean number of taxa observed within the cell. A hierarchical clustering tree was constructed on this set of accumulation patterns, and the optimal number of clusters corresponding to the minimal silhouette width was obtained (column 5) out of the possible number of clusters (number of genera, column 4). These numbers are in good agreement with within-genus taxa accumulation data visualizations.

Dataset	Year	16S segment, Sequencing& Clustering	Pseudo $R^2_{\log\ depth}$	Pseudo $R^2_{\log\ depth+design}$	$AIC_{\log\ depth}$	$AIC_{\log\ depth+design}$
Mouse [1, 2]	2009	V2, 454, FST.97	99.04%	99.14%	1.3853×10^3	1.3230×10^3
Diarrhea [3]	2014	V12, 454, FST.99	99.92%	99.93%	1.2727×10^4	1.2578×10^4
Time series [4]	2014	V4, GAIIx, FST.97	99.95%	99.95%	2.1964×10^3	2.1744×10^3
Wastewater [5]	2018	V34, MiSeq, FST.99	99.94%	99.95%	7.2713×10^2	7.1527×10^2
MBQC-HLB$^{(97)}$ [6]	2017	V4, MiSeq, FST.97	99.83%	99.85%	2.7512×10^3	2.6998×10^3
MBQC-HLB$^{(99)}$ [6]	2017	V4, MiSeq, FST.99	99.98%	99.99%	2.9250×10^3	2.8966×10^3
MBQC-HLB$^{(D)}$ [6]	2017	V4, MiSeq, Dada2	99.67%	99.77%	2.0710×10^3	1.9483×10^3
PIH100$^{(97)}$ [7]	2020	V12, MiSeq, FST.97	99.83%	99.87%	1.3833×10^3	1.3133×10^3
PIH100$^{(99)}$ [7]	2020	V12, MiSeq, FST.99	99.95%	99.95%	1.4208×10^3	1.3853×10^3
PIH100$^{(D)}$ [7]	2020	V12, MiSeq, Dada2	99.46%	99.58%	1.1647×10^3	1.1074×10^3
Pseudomonas$^{(97)}$	2021	V12, MiSeq, FST.97	99.92%	99.95%	3.2197×10^2	2.900×10^2
Pseudomonas$^{(99)}$	2021	V12, MiSeq, FST.99	99.61%	99.98%	3.4318×10^2	3.1313×10^2
Pseudomonas$^{(D)}$	2021	V12, MiSeq, Dada2	99.60%	99.69%	244.77	234.77

Table S2: Relative to study variables, sample depth explains bulk of the systematic variation in a 16S survey's *sample-wide* taxa accumulations. For each 16S survey dataset mentioned in column 1, the year of publication is listed in column 2, the partial 16S segment targeted, machine technology and sequence clustering approach used are specified in column 3. McFadden's pseudo-R^2 for explaining genus-specific taxa accumulations with two negative binomial regressions (NB) are listed in columns 4 and 5. The fourth column is obtained when the NB regression includes logged sampled depth alone as predictor. The fifth column additionally includes the experimental design matrix for each dataset as predictors.

Dataset	Year	16S segment, Sequencing& Clustering	Pseudo $R^2_{\log\ depth}$	Pseudo $R^2_{\log\ depth+design}$	$AIC_{\log\ depth}$	$AIC_{\log\ depth}$
Mouse [1, 2]	2009	V2, 454, FST.97	83.62%	96.69%	1.7443×10^4	1.2576×10^4
Diarrhea [3]	2014	V12, 454, FST.99	87.45%	94.66%	1.8648×10^5	1.5884×10^5
Time series [4]	2014	V4, GAIIx, FST.97	77.52%	97.22%	5.2421×10^4	3.4657×10^4
Wastewater [5]	2018	V34, MiSeq, FST.99	69.45%	90.42%	3.4313×10^4	2.7412×10^4
MBQC-HLB$^{(97)}$ [6]	2017	V4, MiSeq, FST.97	84.28%	92.65%	1.5078×10^5	1.3051×10^5
MBQC-HLB$^{(99)}$ [6]	2017	V4, MiSeq, FST.99	89.15%	94.33%	1.6308×10^5	1.4528×10^5
MBQC-HLB$^{(D)}$ [6]	2017	V4, MiSeq, Dada2	40.72%	73.48%	4.2232×10^4	3.5720×10^4
PIH100$^{(97)}$ [7]	2020	V12, MiSeq, FST.97	80.67%	91.23%	1.3833×10^3	1.3133×10^3
PIH100$^{(99))}$ [7]	2020	V12, MiSeq, FST.99	89.86%	95.10%	1.4210×10^3	1.3853×10^3
PIH100$^{(D)}$ [7]	2020	V12, MiSeq, Dada2	74.48%	82.53%	1.1647×10^3	1.1073×10^3
Pseudomonas$^{(97)}$	2021	V12, MiSeq, FST.97	93.34%	98.54%	4.081×10^3	3.2653×10^3
Pseudomonas$^{(99)}$	2021	V12, MiSeq, FST.99	89.13%	97.70%	4.5456×10^3	3.7133×10^3
Pseudomonas$^{(D)}$	2021	V12, MiSeq, Dada2	75.98%	91.25%	2.2810×10^3	2.0138×10^3

Table S3: Relative to study variables, sample-depth explains bulk of the systematic variation in a 16S survey's *genus-specific* taxa accumulations. For each 16S survey dataset mentioned in column 1, the year of publication is listed in column 2, the partial 16S segment targeted, machine technology and sequence clustering approach used are specified in column 3. McFadden's pseudo-R^2 for explaining sample-wide taxa accumulations with two negative binomial regressions (NB) are listed in columns 4 and 5. The fourth column is obtained when the NB regression includes logged genus recovered abundances alone as predictor. The fifth column additionally includes the experimental design matrix for each dataset as predictors. Six and seventh columns indicate the respective Akaike Information Criteria.

References

[1] Peter J. Turnbaugh, Vanessa K. Ridaura, Jeremiah J. Faith, Federico E. Rey, Rob Knight, and Jeffrey I. Gordon. The effect of diet on the human gut microbiome: a metagenomic analysis in humanized gnotobiotic mice. *Science translational medicine*, 1(6):6ra14–6ra14, 2009. Publisher: American Association for the Advancement of Science.

[2] Joseph Nathaniel Paulson, Nathan D. Olson, Domenick J. Braccia, Justin Wagner, Hisham Talukder, Mihai Pop, and Hector Corrada Bravo. metagenomeSeq: Statistical analysis for sparse high-throughput sequencing, 2021. URL `https://bioconductor.org/packages/metagenomeSeq/`.

[3] Mihai Pop, Alan W Walker, Joseph Paulson, Brianna Lindsay, Martin Antonio, M Anowar Hossain, Joseph Oundo, Boubou Tamboura, Volker Mai, Irina Astrovskaya, Hector Corrada Bravo, Richard Rance, Mark Stares, Myron M Levine, Sandra Panchalingam, Karen Kotloff, Usman N Ikumapayi, Chinelo Ebruke, Mitchell Adeyemi, Dilruba Ahmed, Firoz Ahmed, Meer Taifur Alam, Ruhul Amin, Sabbir Siddiqui, John B Ochieng, Emmanuel Ouma, Jane Juma, Euince Mailu, Richard Omore, J Glenn Morris, Robert F Breiman, Debasish Saha, Julian Parkhill, James P Nataro, and O Colin Stine. Diarrhea in young children from low-income countries leads to large-scale alterations in intestinal microbiota composition. *Genome Biology*, 15(6):R76, 2014. ISSN 1465-6906. doi: 10.1186/gb-2014-15-6-r76. URL `http://www.ncbi.nlm.nih.gov/pmc/articles/PMC4072981/`.

[4] Lawrence A. David, Arne C. Materna, Jonathan Friedman, Maria I. Campos-Baptista, Matthew C. Blackburn, Allison Perrotta, Susan E. Erdman, and Eric J. Alm. Host lifestyle affects human microbiota on daily timescales. *Genome biology*, 15(7):1–15, 2014. Publisher: BioMed Central.

[5] Prachi Kulkarni, Nathan D. Olson, Joseph N. Paulson, Mihai Pop, Cynthia Maddox, Emma Claye, Rachel E. Rosenberg Goldstein, Manan Sharma, Shawn G. Gibbs, Emmanuel F. Mongodin, and Amy R. Sapkota. Conventional wastewater treatment and reuse site practices modify bacterial community structure but do not eliminate some opportunistic pathogens in reclaimed water. *Science of The Total Environment*, 639:1126–1137, October 2018. ISSN 0048-9697. doi: 10.1016/j.scitotenv.2018.05.178. URL `https://www.sciencedirect.com/science/article/pii/S004896971831828X`.

[6] Rashmi Sinha, Galeb Abu-Ali, Emily Vogtmann, Anthony A. Fodor, Boyu Ren, Amnon Amir, Emma Schwager, Jonathan Crabtree, Siyuan Ma, Christian C. Abnet, Rob Knight, Owen White, and Curtis Huttenhower. Assessment of variation in microbial community amplicon sequencing by the Microbiome Quality Control (MBQC) project consortium. *Nature Biotechnology*, 35(11):1077–1086, November 2017. ISSN 1546-1696. doi: 10.1038/nbt.3981. URL `https://www.nature.com/articles/nbt.3981`. Number: 11 Publisher: Nature Publishing Group.

[7] Joseph N. Paulson, Brent L. Williams, Christine Hehnly, Nischay Mishra, Shamim A. Sinnar, Lijun Zhang, Paddy Ssentongo, Edith Mbabazi-Kabachelor, Dona SS Wijetunge, and Benjamin Von Bredow. Paenibacillus infection with frequent viral coinfection contributes to postinfectious hydrocephalus in Ugandan infants. *Science translational medicine*, 12(563), 2020. Publisher: American Association for the Advancement of Science.
