# Supplementary material for: Differential richness inference for 16S rRNA marker gene surveys
Source: Genome Biol. 2022 Aug 1;23:166. doi: 10.1186/s13059-022-02722-x (PMC9344657; doi:10.1186/s13059-022-02722-x)
Supplement: Supplementary file 4 — Additional file 4. Presents a theoretic model for the generation of false species by misclassifying true species in a population survey. Characterizes the influence of false species accumulation on the observed and estimated unobserved richness components underlying standard richness estimators. Related figures and literature references are contained within the note. [file 13059_2022_2722_MOESM4_ESM.pdf]

Additional File 4: The dynamics of false species accumulations in a population survey

Here we study the accumulation dynamics of false species types, generated through misclassification of true species types in a population survey. We incorporate false species types within the sampling theoretic framework of Chao [1] and Harris [2] and study their accumulation behavior analytically and with simulations. An interpretation for the Prokounter's linear predictor proposed in the main manuscript is offered by placing it in the context of observed and asymptotic richness estimates.

Throughout, both the number of true species and the possible false varieties are assumed finite. Thus, we let $i = 1 \dots I$ denote the true number of species with population relative frequencies p_i. Every true species i can be falsely classified as one of $j = 1 \dots J$ species with probabilities $\eta_i q_{ij}$, where η_i is the probability of misclassifying species type i and q_{ij} represents the conditional probability that the misclassification is to a false type j. Then the the probability of sampling a true type i is $p_i(1-\eta_i)$ and the probability of sampling a false type j is $\sum_{i=1}^{I} p_i \eta_i q_{ij}$.

Case 1 We first assume that none of the false species types are identical to any of the true species types. Following Chao [1], we consider the random variable n_r, which is the frequency of frequency r in the observed count data i.e., the number of species registering a count of r. We decompose n_r as a sum of two terms: $n_r = n_r^{(T)} + n_r^{(F)}$, where $n_r^{(T)}$, corresponds to the number of observed true species with frequency r and $n_r^{(F)}$ corresponds to the observed number of false types with frequency r. Let the sampling depth be $N = \sum_r r n_r$. Then, based on eqn. 2 in [1], the average value of n_r specified as a function of sampling depth N can be decomposed as:

$$En_r(N) \approx \underbrace{\sum_{i=1}^{I} \frac{(Np_i(1-\eta_i))^r e^{-Np_i(1-\eta_i)}}{r!}}_{:=\text{expected true species with frequency } r,\ En_r^{(T)}(N)} + \underbrace{\sum_{j=1}^{J} \frac{(N\sum_i p_i\eta_i q_{ij})^r e^{-N\sum_i p_i\eta_i q_{ij}}}{r!}}_{:=\text{expected false types with frequency } r,\ En_r^{(F)}(N)} \tag{1}$$

The total possible false species types J is appropriately recovered at $N = 0$ and $r = 0$:

$$En_0^{(F)}(N=0) = \sum_{j=1}^{J} e^{-N\sum_i p_i\eta_i q_{ij}}|_{N=0} = J. \tag{2}$$

Let $\tilde{J}(N)$ denote the total number of false species types observed when sampling at a depth of N. Thus the expected false species accumulation $E\tilde{J}(N)$ is:

$$\begin{aligned} E\tilde{J}(N) = &\underbrace{En_0^F(0)}_{\text{total false types possible}} - \underbrace{En_0^F(N)}_{\text{average number of as yet undiscovered false types}} \\ &= J - \sum_{j=1}^{J} e^{-N\sum_i p_i\eta_i q_{ij}} \end{aligned} \tag{3}$$

For a given sampling effort N, the total apparent richness (I+J) is decomposed into four categories: number of observed true types ($\tilde{I}(N)$), number of observed false types ($\tilde{J}(N)$), number of undetected true types ($\tilde{I}_u(N)$), number of undetected false types ($\tilde{J}_u(N)$). It is clear from eqn. (3), $E\tilde{J}(N) \to J$ as $N \to \infty$ appropriately indicating that, with sufficient sampling, all possible false discoveries are recovered. Similarly, it can be shown $E\tilde{J}_u(N) \to 0$, $E\tilde{I}(N) \to I$, and $E\tilde{I}_u(N) \to 0$ as $N \to \infty$.Then the average values of observed richness, S^{obs}, follow our intuition:

$$ES^{obs}(N) = E\tilde{I}(N) + E\tilde{J}(N) \to I + J, N \to \infty \tag{4}$$

Thus, without appropriately accounting for false discovery generation, any asymptotically consistent total richness estimator, $\hat{S}(N)$, estimates the total apparent richness of the community as $I+J$, overestimating its true richness.

$$E\hat{S}(N) = E\tilde{I}(N) + E\tilde{J}(N) + E\tilde{I}_u(N) + E\tilde{J}_u(N) + B(N) = I + J + B(N) \to I + J, N \to \infty. \tag{5}$$

where $B(N) \to 0, N \to \infty$ is a bias term.

The Chao1 estimator uses the observed richness $S^{obs}(N)$ to estimate $E\tilde{I}(N)$ and estimates a lower bound for undetected richness with $\frac{En_1^2}{2En_2}$. The two terms are biased by false discoveries as below:

$$\begin{aligned} E\left(S^{obs}(N) - E\tilde{I}(N)\right) &= E\tilde{J}(N) \\ \frac{En_1^2(N)}{2En_2(N)} &= \frac{\left(En_1^{(T)}(N) + En_1^{(F)}(N)\right)^2}{2\left(En_2^{(T)}(N) + En_2^{(F)}(N)\right)} \end{aligned} \tag{6}$$

Appropriate bias correction factors are therefore needed.

Fig. S1 provides a multinomial simulation illustration of the problem setup for various sampling effort N. In the presence of false discoveries, observed and asymptotic richness estimates far exceed true richness and steadily approach the apparent asymptotic richness of the community $I+J$. Furthermore, the sampling depth needed to cover the true community is much lower than what the survey data including false discoveries would indicate.

Case 2 We now allow for the general possibility that true types can also be misclassified to a subset of the true species types. In this case, we decompose the true species types in to two further sub-types: true types that occur because of correct classification and true types that occur because of misclassification from another true type. If J^* represents the number of false types that are not identical to any of the true types, then the total false classifications that could arise are $J = J^* + I$. Eqn. (1) gets adjusted to the following and similar discussions as in Case 1 follow.

$$En_r(N) \approx \underbrace{\sum_{i=1}^{I} \frac{(Np_i(1-\eta_i))^r e^{-Np_i(1-\eta_i)}}{r!}}_{\text{expected true types with freq } r, En_r^{(T)}(N)} + \underbrace{\underbrace{\sum_{i=1}^{I} \frac{(N\sum_{k:k\neq i} p_k \eta_k q_{ki})^r e^{-N\sum_{k:k\neq i} p_k \eta_k q_{ki}}}{r!}}_{\text{expected number of true types because of misclassification at freq. } r} + \sum_{j=1}^{J^*} \frac{(N\sum_i p_i \eta_i q_{ij})^r e^{-N\sum_i p_i \eta_i q_{ij}}}{r!}}_{\text{expected false types at freq. } r, En_r^{(F)}(N)} \tag{7}$$

Case 3 We consider a further simplification to case 1 above. Specifically, we shall assume $\eta_i = \eta$, and all $q_{ij} = q$. Eqn. (1) gets adjusted to:

$$En_r(N) \approx \underbrace{\sum_{i=1}^{I} \frac{(Np_i(1-\eta))^r e^{-Np_i(1-\eta)}}{r!}}_{\text{expected true discoveries with freq. } r,\ En_r^{(T)}(N)} + \underbrace{J\frac{(N\eta q)^r e^{-N\eta q}}{r!}}_{\text{expected false types with freq. } r,\ En_r^{(F)}(N)} \tag{8}$$

Correspondingly, the expected number of false discoveries for a prescribed sampling effort N, from eqn. 3 gets adjusted to:

$$E\tilde{J}(N) = J - Je^{-N\eta q} = J \cdot (1 - e^{-N\lambda}) \tag{9}$$

where $\lambda = \eta q$ is an effective parameter.

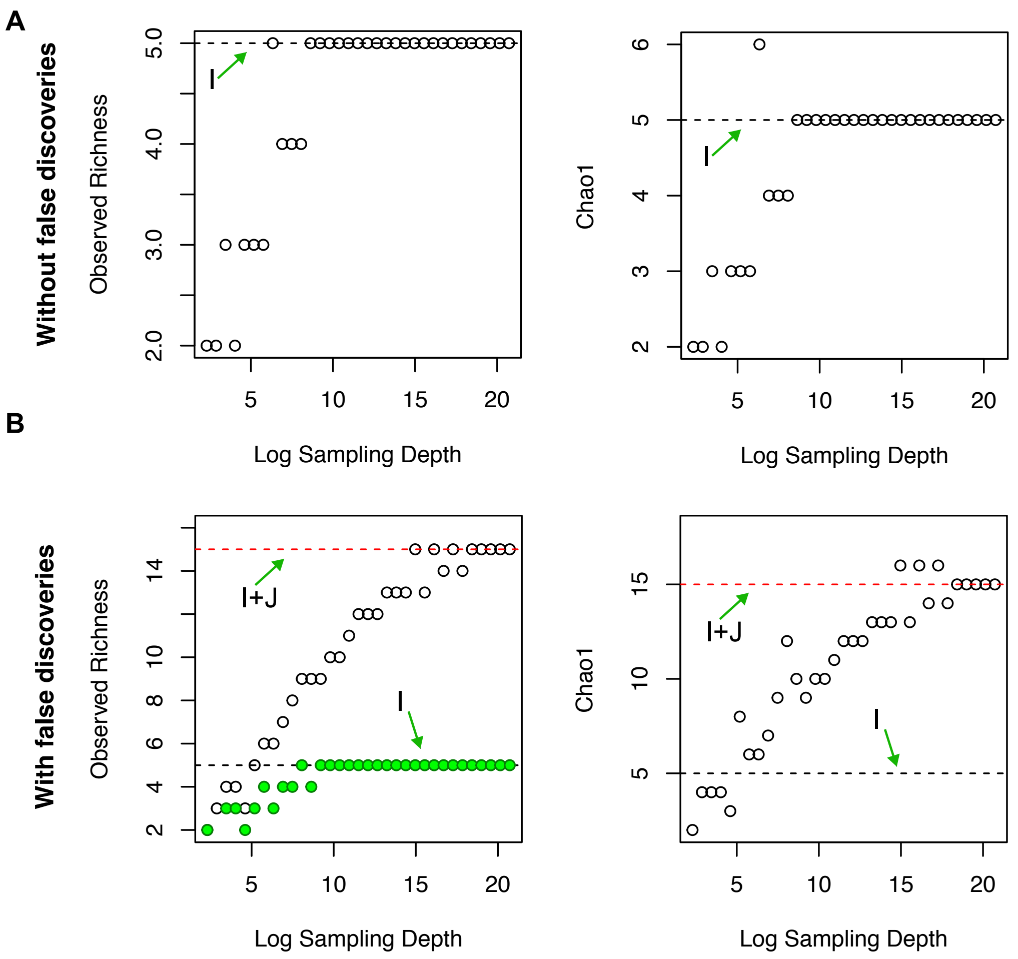

Fig. S1: (A) We construct an artificial in silico community with $I = 5$ true types with $p_i \propto \frac{1}{.1^{i \cdot i}}$, for $i = 1 \ldots 5$. In (B) each of the 5 true types can also be misclassified into to two possible false types with conditional probabilities $q_{i\cdot} \propto (1, 1/2)$ for a total of $J = 10$ false types. Thus the apparent asymptotic richness is $I + J = 15$. Other parameters: $\eta_i = \eta = .01$. Points in green indicate the sampled true richness of the community (i.e., only true species) and the black points indicate the observed apparent richness (true + false species) of the community. In the presence of false discoveries, with sufficient sampling, both the observed and traditional asymptotic richness estimator (here Chao1), overestimate true richness. Observed and asymptotic richness estimates correlate well until the total apparent richness is sufficiently sampled.

.

Fig. S1 motivates the following idea, which is illustrated in Fig. S2. Let N^* denote the sampling effort needed to recover all true species with high probability. Let N^{**} denote the sampling effort needed to recover true *and* all false species types with high probability. With sequencing technologies, it is very likely $N^{**} > N^*$. The sampling effort axis is then split into three windows: $w_1 = [0, N^*]$, $w_2 = (N^*, N^{**})$, $w_3 = [N^{**}, \infty)$.

In the window w_2, the gradient of the expected observed number of features is, with high probability:

$$\frac{dES^{obs}(N)}{dN} = \frac{d\left(E\tilde{I}(N) + E\tilde{J}(N)\right)}{dN} \approx \frac{d(I + E\tilde{J}(N))}{dN} = J \cdot \lambda e^{-N\lambda} \tag{10}$$

from eqn. (9). Thus within window w_2,

$$\log \frac{dES^{obs}(N)}{dN} \approx \underbrace{(\log J + \log \lambda)}_{\text{intercept}} \underbrace{-\lambda}_{\text{slope}} N \tag{11}$$

with high probablity. Then, under this simplified model, a trend line of the log gradient of the expected observed

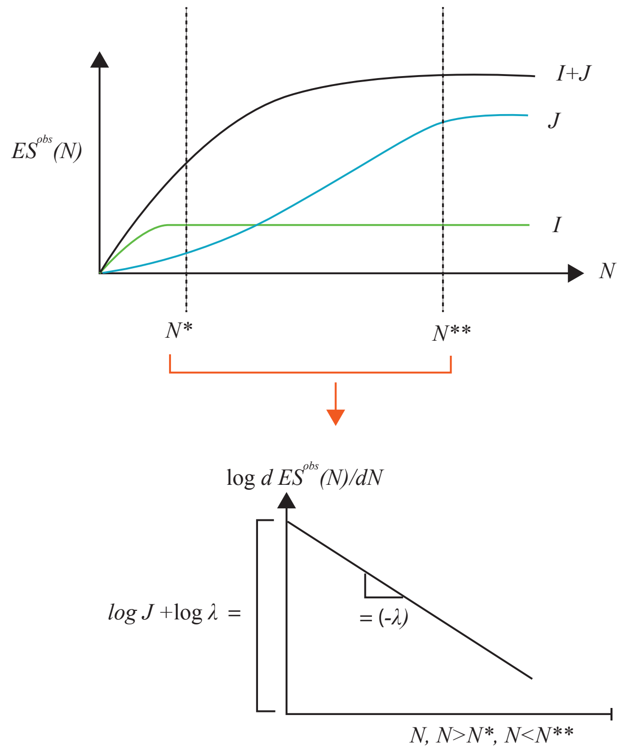

Fig. S2: In the simplified case 3, regressing logged species accumulation rate against sampling effort in the sampling regime $w_2 = (N^*, N^{**})$, yield estimates of asymptotic false richness generated from a survey and the effective species mislassification rate λ.

.

species richness (a rasonable stimate is the log gradient of species rarefaction curve for various $N \in w_2$) against sampling effort N can be used to calculate both the total number of possible false discoveries J, and the net false classification rate λ. Knowledge of these quantities can allow us to establish appropriate corrections for asymptotic richenss estimates and also for quantities that underly species richness calculations.

The problem reduces to identifying w_2, which involves two parameters N^* and N^{**}. If one assumes that practical sampling limitations does not allow us to ever reach N^{**}, N^* alone needs to be estimated. We leave this as an open question and settle for varying N^* through all values N and getting a sense of the range of λ and J values that arise.

Prokounter's proposal

In principle, a finite sampling correction for the observed richness and asymptotic richness estimates is achieved with access to an unbiased estimator $\widehat{E\tilde{J}(N)}$ for $E\tilde{J}(N)$as:

$$\begin{aligned} \widehat{S^{obs}(N)} &= S^{obs}(N) - \widehat{E\tilde{J}(N)};\ E\widehat{S^{obs}(N)} \to I, N \to \infty \\ \widehat{\hat{S}(N)} &= \hat{S}(N) - B(N) - J \approx \hat{S}(N) - B(N) - \widehat{E\tilde{J}(N)};\ E\widehat{\hat{S}(N)} \to I, N \to \infty. \end{aligned} \tag{12}$$

However, in both cases 1 and 2 above, $E\tilde{J}(N)$ is defined based on a number of unknown parameters. In a small survey like a supervised local bird walk where an expert accompanies amateur birders, perhaps I and J are small enough that direct estimation of the average rate parameters are feasible. In large scale automated surveys like 16S surveys, estimation is a challenging problem. The methodology in the main manuscript proposes to circumvent this problem specifically in 16S surveys through a function $f_t(\log N)$ estimated from within-genus taxa accumulation data. Specifically, the

methodology assumes that most within-genus taxa discoveries across detected genera are false. In terms of notation, the main manuscript has the following correspondence for within-genus richness analysis: $N \equiv y_{gj}$, $S^{obs} \equiv n_{gj}$. The linear predictor in the main text models:

$$
\begin{aligned}
\log ES^{obs}(N) &= \log E\left[\tilde{I}(N) + \tilde{J}(N)\right] \\
&= \log E\left[I - \tilde{I}_u(N) + \tilde{J}(N)\right] \\
&= \log\left[I \underbrace{-E\tilde{I}_u(N))}_{\text{sampling effort bias}} + \underbrace{E\tilde{J}(N)}_{\text{false discovery bias}} \right] \\
&\approx \underbrace{X\beta}_{\text{design variables}} + f_t(\log N)
\end{aligned}
\tag{13}
$$

where adjustments for false discovery accumulation and sampling effort N are folded into $f_t(\cdot)$ and any additional predictors we may wish to incorporate. When asymptotic richness estimates are used as response variables, a similar interpretation follows involving the bias term $B(N)$ in eqn. (12).

References

[1] Chao, A. Nonparametric Estimation of the Number of Classes in a Population. *Scandinavian Journal of Statistics* **11**, 265–270 (1984). URL `https://www.jstor.org/stable/4615964`. Publisher: [Board of the Foundation of the Scandinavian Journal of Statistics, Wiley].

[2] Harris, B. Determining bounds on integrals with applications to cataloging problems. *The Annals of Mathematical Statistics* 521–548 (1959). Publisher: JSTOR.
