# Supplementary material for: Differential richness inference for 16S rRNA marker gene surveys
Source: Genome Biol. 2022 Aug 1;23:166. doi: 10.1186/s13059-022-02722-x (PMC9344657; doi:10.1186/s13059-022-02722-x)
Supplement: Supplementary file 5 — Additional file 5. Presents simulation details for studying the confounding introduced by between-sample differential abundance of detected taxa in between-sample differential richness. [file 13059_2022_2722_MOESM5_ESM.pdf]

Additional File 5 : Simulations

To illustrate that skewed differential abundance of genera can lead to confounded sample-wide differential richness inference with current estimators, we built the following two-group simulator. The number of noisy taxa generated for a given genus is derived as a function of genus abundance .

The simulation procedure is executed independently for each sample-group, and genus category. We therefore do not explicitly indicate the genus and sample group in the notation below. j indexes observations, i indicates the true types within the genus, k indexes the set of taxa (artifactual + 1 true type) generated from the true type. The recovered abundance for a taxon is indicated as y_{jik}. $+$ in a subscript index indicates summing over that index.

$$\begin{aligned}
& y_{jik}|y_{ji+},\theta_{i\cdot} \sim Multinomial(y_{ji+},\theta_{ji}) \\
& \theta_{ji} \sim Dirichlet(1,\frac{1}{2},\frac{1}{3},\cdots\frac{1}{\tilde{n}_{ji}}) \\
& \tilde{n}_{ji}|y_{ji+} = \lceil e^{f_t(y_{ji+})} \rceil \\
& y_{ji+}|y_{j++},\nu_{j\cdot} \sim Multinomial(y_{j++},\nu_{j\cdot}), \nu_{ji} \propto u_{ji} \\
& u_{ji} \sim Uniform(0,1), i = 1 \ldots n_j^0
\end{aligned} \tag{1}$$

In words, a given genus-wide recovered abundance y_{j++} is distributed among its constituent biological types (species) as y_{ji+}. Each species-specific abundance is further distributed among all the taxa (true + artifactual taxa) constituting the species as y_{jik} for $k = 1 \ldots \tilde{n}_i$. The number of taxa generated for a true species type $\tilde{n}_{ji}$ is a function of its recovered abundance y_{ji+} for the species. The Dirichlet parameter is set such that on average, the first category is the dominant, corresponding to the true taxon for the type. The rest are artifactual. For the null case, the true richness n^0 are input parameters, are not varied for samples within a group. Genus-wide recovered abundances y_{j++} are sampled from any given real dataset. The median genus-wide recovered abundances were used as a threshold to define "low" and "high" values for inducing differential abundance. Additional Guassian noise is injected in the sampled recovered abundance values. $f_t(\cdot)$ is a fitted loess trend for within-genus taxa accumulation data.
