# Supplementary material for: Differential richness inference for 16S rRNA marker gene surveys
Source: Genome Biol. 2022 Aug 1;23:166. doi: 10.1186/s13059-022-02722-x (PMC9344657; doi:10.1186/s13059-022-02722-x)
Supplement: Supplementary file 6 — Additional file 6. Presents details on smoothing spline model construction. Related literature references are contained within the note. [file 13059_2022_2722_MOESM6_ESM.pdf]

# Additional File 6: SSANOVA model construction

Here, we present more details on the construction of the smoothing spline ANOVA model specified in equation (1) of the main text. We assumed for $z_{gj} = \log n_{gj}$:

$$E[z_{gj}|g,y_{gj}] = \eta(g,y_{gj}) + \varepsilon_{gj} = \kappa + f_R(\log y_{gj}) + f_{GR}(g,\log y_{gj}) + \varepsilon_{gj},\ \varepsilon_{gj} \sim N(0,\sigma^2)$$

Here, $\eta$ is assumed a member of a reproducing kernel Hilbert space (RKHS) of functions $H$ with the orthogonal decomposition $H = H_0 \oplus H_1$. $H_0$ is a finite dimensional space spanned by a set of basis functions $\phi_1(\cdot),\ldots,\phi_m(\cdot)$. $H_1$ is an RKHS with an associated bivariate, symmetric reproducing kernel $R_1(\cdot,\cdot)$. Suppose we wish to model a given set of responses $\tilde{y}_1,\ldots,\tilde{y}_n$, through a smooth function $\eta$ over predictors $x_1,\ldots,x_d$. It can be shown that $\eta(x_i)$ is conveniently decomposed orthogonally as $\eta(\cdot) = \eta_0(\cdot) + \eta_1(\cdot) + \rho(\cdot)$, with $\eta_0(\cdot) \in Span(\{\phi_i, i = 1\ldots m\})$, $\eta_1(\cdot) \in Span(\{R_1(x_i,\cdot), i = 1\ldots n\})$ and $\rho(\cdot) \in H_1 \ominus Span(\{R_1(x_i,\cdot), i = 1\ldots n\})$.

For estimating $\eta$, consider the penalized optimization problem:

$$\hat{\eta} = \arg\min_{h\in H} l(h|\tilde{y}.,x.) + \lambda J(h), \tag{E1}$$

where $l(\cdot|\tilde{y}.,x.)$ is the negative log likelihood, $J(\cdot)$ is a roughness penalty, and $\lambda$ a regularization parameter. $J(h)$ is a squared semi-norm in $H_1$ and penalizes overfitting $h$ to data in $H_1$. The celebrated Kimeldorf-Wahba[1] theorem proves that the solution to this penalized optimization problem has a finite dimensional representation:

$$\hat{\eta}(\cdot) = \sum_{i=1}^{m} c_i\phi_i(\cdot) + \sum_{i=1}^{n} r_i R_1(x_i,\cdot) \tag{E2}$$

This allows one to write $J(h) = r_.^T R_1 r.$ , where the $(i,j)^{th}$ element of $R_1$ is $R_1(x_i,x_j)$. As an example, a cubic smoothing spline on $[0,1]$ is obtained as solution to the following instance of the penalized optimization problem in eqn. (E1): $\tilde{y}_i = \eta(x_i) + \varepsilon_i, \varepsilon_i \sim N(0,\tau^2), x_i \in [0,1], J(\eta) = \int_0^1 \left(\frac{d^2\eta}{dx^2}\right)^2 dx.$

Construction of the recovered abundance dependent model in eqn. (1) in the main text proceeds as follows. As per example 2.7 in Gu[2], we specify marginal reproducing kernels for (A) the discrete space of genera $\{1\ldots K\}$, where $K$ denotes the number of detected genera, and (B) the continuous space of $\log y_{gj}$ rescaled to $[0,1]$ via the transformation $t_{gj} = \frac{y_{gj} - max(y_{..})}{max(\log y_{..}) min(\log y_{..})}$. Reproducing kernels for the product space $\{1\ldots K\} \otimes [0,1]$ - decomposed in to orthogonal subspaces corresponding to the intercept, genus-specific main effects term, (rescaled) recovered abundance dependent main effects term, and the interaction terms - are constructed as tensor products of marginal reproducing kernels. These are derived in example 2.7 and presented in table 2.5, Gu[2]. The intercept and any linear dependence on the $t_{gj}$ are members of $H_0$, spanned by the basis $\{\phi_1(x) = 1, \phi_2(x) = x - 0.5\}$, $x \in [0,1]$. The higher order terms are penalized for roughness and are elements of $H_1$, the reproducing kernel $R_1(\cdot,\cdot)$ of which is constructed using table 2.5, Gu[2]. Specifications of $\phi_.(\cdot)$ and $R_1(\cdot,\cdot)$ then enter the optimization problem in eqn. (E1) where $h$ has the form specified in eqn. (E2). To estimate $\kappa$, $f_R(\cdot)$ and $f_{GR}(g,\log y_{gj})$ using the `gss` package, we pass the R model formula $z_{gj} \sim\ g * log(y_{gj})$ to the `gss::ssanova` function.

## References

[1] George Kimeldorf and Grace Wahba. Some results on Tchebycheffian spline functions. *Journal of mathematical analysis and applications*, 33(1):82–95, 1971. Publisher: Elsevier.

[2] Chong Gu. *Smoothing spline ANOVA models*, volume 297. Springer Science & Business Media, 2013.
